# *Lactobacillus plantarum* and *Bacillus licheniformis* Ameliorate High‐Carbohydrate–High‐Fat‐Induced Glycolipid Disorders in Common Carp in Association With the Gut Microbiota and Bile Acid Axis

**DOI:** 10.1155/anu/6145147

**Published:** 2026-09-29

**Authors:** Peng Pang, Xiao Yan, Xin Long, Jiali Mi, Chaobin Qin, Liping Yang, Guoxing Nie

**Affiliations:** ^1^ College of Fisheries Henan Normal University No. 46 Jianshe Road Xinxiang 453007 Henan Province China henannu.edu.cn

**Keywords:** bile acid metabolism, common carp (*Cyprinus carpio*), glycolipid metabolism, gut microbiota, high-carbohydrate-high-fat diet, probiotics

## Abstract

High‐carbohydrate–high‐fat (HCHF) diets help reduce feed costs in intensive aquaculture, but they often induce glycolipid metabolic disorders and hepatopancreatic steatosis in farmed fish. Although probiotics have shown promise in ameliorating metabolic disorders, the mechanisms by which they interact with the gut microbiota–bile acid (BA) axis in fish remain poorly understood. In this study, common carp were fed an HCHF diet to establish a glycolipid disorder model, followed by dietary supplementation with *Lactobacillus plantarum* (LP) and *Bacillus licheniformis* (BL) for 8 weeks. Multiomics approaches, including *16S* rRNA sequencing, targeted BA metabolomics, and untargeted intestinal metabolomics, were integrated with phenotypic, biochemical, histomorphological, and molecular analyses. HCHF feeding significantly promoted growth but was associated with systemic glycolipid dysfunction. Probiotic supplementation improved glycolipid profiles and antioxidant capacity, alleviated hepatopancreatic lipid accumulation and damage, and activated the hepatic farnesoid X receptor (FXR)/small heterodimer partner (SHP) signaling pathway. Both probiotics reshaped the gut microbiota by enriching the fish‐specific beneficial bacterium *Cetobacterium somerae* and taxa with potential bile salt hydrolase activity, while also modulating key intestinal metabolic pathways. Notably, BL exerted a more pronounced regulatory effect on BA profiles. These findings suggest that LP and BL alleviate HCHF‐induced glycolipid disorders in common carp, with the gut microbiota–BA axis serving as a potential key regulatory node, providing a theoretical basis for probiotic application in functional aquafeeds.

## 1. Introduction

Fish provide a vital source of animal protein for human consumption. Global marine fishery resources have been declining since the 1970s due to environmental pollution and overfishing [1]. Aquaculture has therefore emerged as an alternative to alleviate pressure on wild fish stocks. As stated in the 2024 edition of *The State of World Fisheries and Aquaculture* released by the United Nations Food and Agriculture Organization, global aquaculture output outpaced that of capture fisheries for the first time in 2022, reaching 130.9 million tons. This figure included 94.4 million tons of aquatic animal products, making aquaculture the world’s leading source of such products [2]. Nevertheless, the scarcity and high cost of high‐quality feed protein sources pose significant challenges to the aquaculture industry [3]. Consequently, the incorporation of readily available and low‐cost carbohydrates and lipids into the diet has become an effective strategy for conserving protein resources.

Carbohydrates and lipids serve as the primary nutrients and energy sources for the growth of fish. An appropriate balance of carbohydrates and fats in feed can improve fish growth performance [4, 5]. Excessive carbohydrates may reduce fish growth rates and digestibility, leading to elevated blood glucose (Glu) levels and excessive glycogen accumulation. Surplus carbohydrates may also be converted into lipids, which accumulate in significant quantities within tissues such as the liver and can ultimately lead to liver enlargement. Conversely, elevated levels of fat in feed can promote fish growth; however, they may also induce significant fat deposition, precipitate nutritional fatty liver, cause glycolipid metabolism disorders, and impair immunity and antioxidant capacity [6, 7]. Investigations concerning juvenile turbot (*Scophthalmus maximus*) have indicated that high‐carbohydrate diets reduce growth performance and lead to abnormal carbohydrate metabolism levels [8]. Furthermore, high‐lipid diets negatively impact the growth performance and feed utilization rate of abalone (*Haliotis discus hannai*) while promoting oxidative stress, inflammation, and apoptosis [9]. Clearly, diets rich in fats and carbohydrates have a major impact on fish health. A key goal of aquaculture research is therefore to understand and mitigate the adverse effects of high‐carbohydrate and high‐fat diets on farmed fish.

There is a substantial anatomical and physiological relationship between the liver and the gut. This concept of the “gut–liver axis,” first proposed by Marshall in 1998, emphasizes the close anatomical and functional connections between the gut and the liver [10]. Subsequent studies have extended this concept, demonstrating bidirectional interactions between these organs in substance transport, energy metabolism, and immune regulation through the portal vein system [11–13]. Glycolipid metabolism is a key part of the gut–liver axis. Studies on mammals have demonstrated that lipids and carbohydrates are converted in the gut into fatty acids and Glu, which are subsequently absorbed into the bloodstream. Excessive consumption of carbohydrates or abnormal insulin function may contribute to disorders of glycolipid metabolism, associated with fat deposition and the development of metabolic dysfunction‐associated fatty liver disease (MAFLD). However, compared with mammals, fish exhibit a diminished capacity to utilize carbohydrates, and this ability varies among distinct fish species [14]. Consequently, the investigation of carbohydrate metabolism in fish is more complex than that in mammals. Additionally, the liver is essential for bile acid (BA) synthesis and cholesterol metabolism, which influences the balance of lipid metabolism. Certain microorganisms present in the animal gut can secrete BSH. BSH hydrolyzes conjugated BAs that enter the gut into free BAs, which are subsequently dehydroxylated by 7α‐dehydroxylase to produce secondary BAs [15, 16]. Within the gut, secondary BAs are reabsorbed into the liver.

The enterohepatic circulation of BAs relies on the regulation of the farnesoid X receptor (FXR). BAs serve as endogenous ligands for the FXR [17, 18]. When FXR is activated, intestinal fibroblast growth factor 15/19 (FGF15/19) and hepatic small heterodimer partner (SHP) are induced, resulting in a negative feedback process that controls the synthesis and transit of BAs [19]. Fish have been found to have a similar process. In the liver of rainbow trout (*Oncorhynchus mykiss*), SHP inhibits BA synthesis by downregulating the expression of BA synthesis‐related genes, particularly cholesterol 7α‐hydroxylase (CYP7A1) and sterol 12α‐hydroxylase (CYP8B1) [20]. FXR acts as a key target in maintaining glycolipid metabolism homeostasis [21]. The activation of hepatic FXR regulates gluconeogenesis and glycogen synthesis while enhancing insulin sensitivity [22–24]. Furthermore, FXR modulates triglyceride (TG) and cholesterol metabolism through various pathways [25]. Consequently, FXR offers new perspectives for regulating blood lipid balance, in addition to treating type 2 diabetes mellitus (T2DM) and MAFLD. In mammals, probiotics modulate glycolipid metabolism via the gut microbiota–BA axis. In fish, probiotics have also shown beneficial effects on health and metabolism, yet their regulatory mechanisms through this axis in common carp fed a high‐carbohydrate–high‐fat (HCHF) diet remain largely unexplored.

The common carp is a highly esteemed freshwater fish species in Northern China, valued for its rapid growth rate, environmental adaptability, and culinary significance. However, the rapid development of intensive aquaculture has introduced challenges such as high‐stocking densities, frequent feeding, and prolonged consumption of high‐energy feeds. These conditions have led to increased disease incidence and excessive lipid deposition, both of which are closely associated with metabolic disorders. Among various probiotic candidates, *Lactobacillus plantarum* (LP) and *Bacillus licheniformis* (BL) stand out as two of the most extensively studied species in aquaculture, yet they operate through fundamentally different mechanisms. LP produces organic acids and bacteriocins that lower intestinal pH and inhibit pathogens [26]; BL secretes digestive enzymes and consumes oxygen, creating a favorable environment for anaerobic beneficial bacteria [27]. In common carp, dietary LP has been reported to enhance growth and immune resistance [28], while BL improves growth, antioxidant status, and intestinal barrier function [29]. Despite these documented benefits, the pathways through which these probiotics regulate BA metabolism and interact with the gut microbiota under HCHF‐induced metabolic stress remain unclear, particularly regarding the gut microbiota–BA–FXR axis in common carp. To address this, we established an HCHF‐induced glycolipid disorder model and used multiomics approaches to investigate the underlying mechanisms linking gut microbiota, BA metabolism, and host metabolic regulation.

## 2. Materials and Methods

### 2.1. Ethics Statement

This study was approved by the Institutional Animal Care and Use Committee of Henan Normal University and carried out in compliance with the “Guidelines for the Ethical Treatment of Experimental Animals” (Approval Number HNSD‐2026BS‐0303).

### 2.2. Experimental Diets and Design

All dry ingredients were ground and passed through a 360‐μm sieve. Fish oil and distilled water were then added, and the mixture was homogenized thoroughly. The mixture was then extruded through an F‐26 twin‐screw extruder equipped with a 1.5 mm‐diameter die to form sinking feed pellets. The resulting feed particles were dried and preserved in a dry, well‐ventilated environment until use. The formulation of the raw materials necessary for the experiment is presented in Supporting Information Table S1.

The experiment was conducted at the Zhongkang Aquaculture Base of Yuanhui District, Luohe City, Henan Province, via the pond cage culture model.

To establish and validate an HCHF metabolic disorder model in common carp and ensure rigorous probiotic interventions, this study first performed a model‐building trial. In the first trial, 180 common carp, weighing 7.43 ± 1.40 g, were randomly divided into six cages. These cages included a CON group (control) with 6.43% lipids and 26.8% carbohydrates and an HCHF model group comprising 12.78% lipids and 40% carbohydrates, with 30 fish per cage and three duplicates per group.

In the second trial, 270 common carp, weighing 47.90 ± 5.84 g, were randomly allocated to nine cages, with three replicates per group and 30 fish per cage. The HCHF diet served as the control, and two probiotic‐supplemented groups (HCHF + LP and HCHF + BL) were established based on this model. LP strain LP194 (CGMCC 21766) was provided by Luohe Wecare Probiotics Biotechnology Co., Ltd., and BL strain (ACCC 01649) was supplied by Henan Jinbaihe Biotechnology Co., Ltd. LP LP194 exhibits high‐acid tolerance and bile salt tolerance [30, 31], while BL ACCC 01649 produces amylase and B‐complex vitamins in the intestine, and its aerobic, spore‐forming nature allows it to deplete intestinal oxygen, thereby establishing an anaerobic niche that favors the growth of anaerobic beneficial bacteria [32]. The identity and purity of both commercial strains are certified by their deposition in the national culture collections (CGMCC and ACCC) and by the manufacturers’ documentation. Both probiotics had a guaranteed viability of 1 × 10^11^ CFU g^−1^. The powders were added to the HCHF diet at a mass ratio of 1:1000 to achieve a final theoretical concentration of 1 × 10^8^ CFU g^−1^ feed. Fresh feed was prepared every 3 days and stored at 4°C to minimize viability loss. During storage, the viable counts of both probiotics were monitored daily.

Prior to the experiment, the fish were subjected to artificial feeding and acclimation for 2 weeks. The fish were fed three times daily at 7:30, 12:00, and 17:30 during acclimation. The feeding regimen during the two 8‐week experimental periods was the same as that during acclimatization. The water in the pond was kept at 27 ± 3°C, and the pH was stable at ~7.2.

### 2.3. Sample Collection and Analysis

In common carp, as in other cyprinids, the pancreas is diffused and embedded within the liver parenchyma, forming a compound organ (the hepatopancreas) that cannot be separated by gross dissection. Accordingly, the tissue collected for all analyses in this study is the hepatopancreas. Prior to being sampled, the fish were fasted for 24 h. To minimize stress prior to sampling, fish were anesthetized with 55 mg L^−1^ MS‐222 (Shanghai Aladdin, China). All samples were assigned random codes during collection to enable blinded analysis. Twelve fish per group were selected for growth index assessment. Blood samples were extracted from the tail vein and centrifuged at 4°C and 3500 ×*g* for 10 min, and the extracted serum was stored at −80°C for subsequent use. The gut and hepatopancreas tissue samples were quickly dissected in a sterile setting, flash‐frozen in liquid nitrogen, and maintained at −80°C for RNA extraction. The leftover gut and hepatopancreatic portions were retained for enzyme activity tests. Three fish per group were chosen for the collection of intact hepatopancreas tissues, which were fixed in 4% paraformaldehyde for histomorphology analysis. BA‐targeted metabolomics, western blotting, and nutritional composition were performed on additional hepatopancreas samples that were frozen in liquid nitrogen and stored at −80°C. To analyze the gut microbiota and detect untargeted metabolites, the intestinal contents were collected.

### 2.4. Growth Performance

The growth and feed utilization parameters were calculated as described in the Supporting Information.

### 2.5. Nutrient Composition

Following established procedures, the contents of ash, crude protein, crude lipid, and dry matter were ascertained. The moisture content was ascertained by drying the samples in an oven at 105°C until their weight remained constant. After acid digestion, the Kjeldahl technique was used to estimate the crude protein. Petroleum ether was used for crude lipid extraction. The samples were burned in a muffle furnace at 550°C for 8 h to determine the amount of ash present. Weighing the material before and after treatment allowed us to determine the amounts of moisture, ash, and crude lipids.

### 2.6. Enzyme Activities and Biochemical Assays

Glu was quantified with commercial kits purchased from BIOSINO (China). TG, total cholesterol (T‐CHO), high‐density lipoprotein cholesterol (HDL‐C), low‐density lipoprotein cholesterol (LDL‐C), total BA (TBA), malondialdehyde (MDA), alanine aminotransferase (ALT), aspartate transaminase (AST), total superoxide dismutase (T‐SOD), lactate (LD), and catalase (CAT) were measured via commercial diagnostic kits obtained from Nanjing Jiancheng Bioengineering Institute (China). The activities of hexokinase (HK), pyruvate kinase (PK), phosphoenolpyruvate carboxykinase (PEPCK), and glucose‐6‐phosphatase (G6Pase) were determined via commercial biochemical kits from Solarbio (China). Catalog information is available in Supporting Information Table S2.

### 2.7. Total RNA Extraction and Quantitative Real‐Time PCR

Total RNA from the hepatopancreas and gut was extracted with an RNAiso Plus Kit (Takara Bio, Japan), and reverse transcription was conducted following the instructions of the PrimeScript RT Reagent Kit (Takara Bio, Japan). RT‐PCR was carried out with a ChamQ Universal SYBR qPCR Master Mix (Vazyme, China) on a LightCycler 480 instrument (Roche, Switzerland). Supporting Information Table S3 contains the primer sequences for the target genes. *18S* ribosomal RNA was used as the reference gene in the 2^–ΔΔCT^ technique to evaluate the relative expression of target genes.

### 2.8. Western Blotting

The proteins were extracted from the hepatopancreas via RIPA lysis buffer supplemented with protease and phosphatase inhibitors (50:1, Solarbio, China). The protein content was determined via a BCA kit (Solarbio, China). Following denaturation via SDS‒PAGE, the proteins were deposited onto 0.45 μm thick PVDF membranes. After the membrane was incubated for one and a half hours with TBST buffer containing 5% BSA, it was incubated with an HRP‐conjugated secondary antibody at room temperature for 2 h. The bands were then visualized via enhanced chemiluminescence (ECL) reagent via a C400 instrument, and quantification was performed via ImageJ software (USA). For western blotting, antibodies against FXR and SHP (1:1000, BIOSS, China) and phospho‐AMPKα, and AMPKα (1:1000, CST, USA) were used, with total proteins being internally referenced by β‐actin (1:20000, Proteintech, China). Antibody information is detailed in Supporting Information Table S4.

### 2.9. Histomorphology Observation

Hepatopancreas tissues were sectioned at 4 μm for hematoxylin‒eosin (H&E) staining (Solarbio, China), embedded in paraffin, and fixed with 4% paraformaldehyde. The samples fixed in paraformaldehyde were dehydrated with 10%, 20%, and 30% sucrose gradients, embedded in OTC, and sectioned into frozen sections. The samples were examined under an optical microscope after being stained with oil red O (Solarbio, China). For histological evaluation, multiple random fields of view were selected from each section to ensure an unbiased assessment.

### 2.10. Analysis of the Gut Microbiota

An OMEGA soil DNA kit (Omega Bio‐Tek, M5635‐02, USA) was used to extract DNA from the intestinal content samples. The forward primer 27F (5′‐AGAGTTTGATCMTGGCTCAG‐3′) and reverse primer 1492R (5′‐ACCTTGTTACGACTT‐3′) were used to amplify the full‐length bacterial *16S* rRNA gene via PCR. Following the purification of the PCR products, fluorescence quantitative analysis was conducted with a microplate reader (BioTek, FLx800) and a Quant‐iT PicoGreen dsDNA Assay Kit (Invitrogen, P7589). Community DNA fragments were sequenced via single molecule real‐time (SMRT) technology on the PacBio Sequel III platform (Shanghai Personal Biotechnology Co., Ltd.). The data were analyzed via QIIME2 and processed with DADA2 [33] for primer trimming, quality filtering, denoising, assembly, and chimera removal to generate amplicon sequence variants (ASVs). The length distribution of high‐quality sequences was subjected to statistical analysis. Taxonomic annotation and diversity analysis were performed via the Greengenes database [34], with species differences and marker species examined. Metabolic pathways were analyzed via the Kyoto Encyclopedia of Genes and Genomes (KEGG, http://www.genome.jp/kegg/pathway.html).

### 2.11. Detection of Untargeted Metabolites in Intestinal Contents

A prechilled methanol/acetonitrile/water mixture (2:2:1, v/v) was used. The intestinal contents were gently thawed at 4°C, vortexed, and treated with low‐temperature ultrasonication for 30 min. The mixture was incubated at −20°C for 10 min and then centrifuged at 14,000 ×*g* and 4°C for 20 min. The supernatant was vacuum‐dried, reconstituted in 100 μL of acetonitrile/water (1:1, v/v), vortexed, and centrifuged again at 14,000 ×*g* and 4°C for 15 min. The final supernatant was collected for LC‐MS/MS analysis.

Analyses were performed via an Agilent 1290 Infinity LC UHPLC system coupled with an AB Sciex TripleTOF 6600 quadrupole time‐of‐flight mass spectrometer at Shanghai Personal Biotechnology Co., Ltd. HILIC separation was carried out on an ACQUIY UPLC BEH amide column (2.1 mm × 100 mm, 1.7 µm; Waters, Ireland). Electrospray ionization was performed in both positive and negative ion modes. The autosampler was maintained at 4°C.

The samples were analyzed in random order to minimize detection variation, and quality control (QC) samples were inserted to assess the reliability and stability of the system.

After data normalization, multivariate statistical analyses (PCA and OPLS‐DA) were performed via the R package ropls. Sevenfold cross‐validation and response permutation testing were used to evaluate model robustness. Variable importance in projection (VIP) values were applied to estimate the contribution of each variable to the OPLS‐DA model.

### 2.12. BA‐Targeted Metabolomics of the Hepatopancreas

After ball‐milling, 20 mg of each tissue sample was extracted with 200 μL of methanol/acetonitrile (v/v = 2:8). Then, 10 μL of mixed internal standard (IS) solution (1 μg mL^−1^) was added as an IS for quantification. The mixture was kept at −20°C for 10 min to precipitate proteins, followed by centrifugation at 12,000 rpm and 4°C for 10 min. The supernatant was transferred to sterile microcentrifuge tubes and vacuum‐dried. The dried residues were redissolved in 100 μL of 50% (v/v) methanol, and the reconstituted solution was used for subsequent analyses.

Sample extracts were analyzed via an LC‐ESI‐MS/MS system consisting of a Sciex UHPLC ExionLC AD system and an Applied Biosystems 6500 Triple Quadrupole mass spectrometer. Chromatographic separation was performed on a Waters ACQUITY UPLC HSS T3 C18 column (100 mm × 2.1 mm, 1.8 µm). Mobile phase A was ultrapure water with 0.01% acetic acid and 5 mmol L^−1^ ammonium acetate; mobile phase B was acetonitrile with 0.01% acetic acid. The gradient elution program was as follows: 0–0.5 min: B increased from 5% to 40%; 0.5–4.5 min: B increased to 50%; 4.5–7.5 min: B increased to 75%; 7.5–10 min: B increased to 95%; 10–12 min: 95% B isocratic; and 12–14 min: B returned to 5%. The flow rate was 0.35 mL min^−1^, the column temperature was 40°C, and the injection volume was 3 μL. The column eluent was directly analyzed via an ESI‐QTRAP mass spectrometer.

Mass spectrometric analysis was performed on a QTRAP 6500+ LC‐MS/MS system equipped with an ESI TurboIon‐Spray interface operating in the negative ionization mode and controlled by Analyst 1.6.3 software (Sciex). The key ESI parameters were as follows: source temperature, 550°C; ISP, −4500 V; and curtain gas (CUR), 35 psi. BAs were quantified via scheduled multiple reaction monitoring (MRM). The declustering potential (DP) and collision energy (CE) were optimized for each MRM transition.

Data acquisition and metabolite quantification were performed via Analyst 1.6.3 and Sciex MultiQuant 3.0.3 software, respectively. Specific MRM transitions were monitored according to the elution time window of each metabolite. PCA and OPLS‐DA were performed on the BA profiles. Differentially abundant metabolites were identified on the basis of VIP values from the OPLS‐DA model and *p*‐values from univariate analysis.

### 2.13. Statistical Analysis

Prior to statistical analysis, data normality and homogeneity of variance were examined. Differences between the CON and HCHF groups were analyzed via independent‐samples *t*‐tests, whereas one‐way ANOVA was used for the probiotic‐supplemented groups. The data are presented as the means ± SEM. Statistical analyses were performed via SPSS 26.0 (IBM, Chicago, USA). Differences were considered significant at *p* < 0.05. Asterisks ( ^∗^) denote significant differences according to the *t*‐test, and different lowercase letters indicate significant differences according to one‐way ANOVA. Metabolites with VIP > 1 and *p* < 0.05 were identified as significantly altered. Spearman’s correlation analysis was used for the microbiota data, and Pearson’s correlation analysis was used for the metabolome and BA profiles.

## 3. Results

### 3.1. The HCHF Diet Promoted Growth and Induced Metabolic Disorders in Common Carp

Supporting Information Table S5 displays the growth performance. The final body weight, weight gain rate, specific growth rate, feed conversion ratio, and hepatosomatic index were significantly greater in the HCHF group than in the CON group (*p* < 0.05). Furthermore, compared with those in the CON group, the crude lipid contents in the muscles, hepatopancreas, and whole body were significantly greater in the HCHF group (*p* < 0.05, Figure 1A). Compared with those in the CON group, the serum levels of GLU, HDL‐C, LDL‐C, T‐CHO, ALT, and MDA in the HCHF group were significantly greater, whereas the activities of T‐SOD and CAT were significantly lower (*p* < 0.05, Figure 1B–G). In the hepatopancreas, the levels of LDL‐C and the activity of T‐SOD were significantly decreased, whereas the levels of TBA, TG, AST, and MDA were significantly elevated (*p* < 0.05), with no significant change in T‐CHO levels (*p* > 0.05). Additionally, the activity of PEPCK, a key gluconeogenic enzyme in the hepatopancreas, was significantly increased (*p* < 0.05, Figure 1H–N). The abdominal cavity anatomy, Oil Red O staining, and H&E staining results are presented in Figure 2A. Extensive abdominal fat deposition was observed in the HCHF group. Compared with those in the CON group, hepatocytes in the HCHF group were disordered in their arrangement, with hepatocyte vacuolization and nuclear displacement. Furthermore, lipid droplets were more prevalent in the hepatopancreatic tissues of the HCHF group.

**Figure 1:**
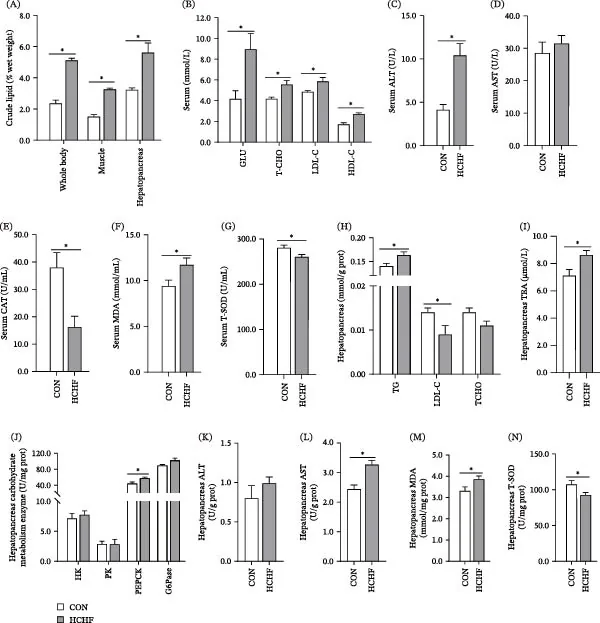
Characterization of glycolipid metabolism disorders induced by the HCHF diet in common carp. (A) Crude lipids from the whole body, muscle, and hepatopancreas. (B) Serum Glu, T‐CHO, LDL‐C, and HDL‐C levels. (C) Serum ALT. (D) Serum AST. (E) Serum CAT. (F) Serum MDA. (G) Serum T‐SOD. (H) Hepatopancreas TG, LDL‐C, and T‐CHO. (I) Hepatopancreatic TBA. (J) Hepatopancreatic carbohydrate metabolism enzymes: HK, PK, PEPCK, and G6Pase. (K) Hepatopancreas ALT. (L) Hepatopancreas AST. (M) Hepatopancreas MDA. (N) Hepatopancreatic T‐SOD. All the data are presented as the means ± SEM (nutrient composition, *n* = 8; enzyme activities and biochemical assays, *n* = 8;  ^∗^ *p* < 0.05;  ^∗∗^ *p* < 0.01; and  ^∗∗∗^ *p* < 0.001).

**Figure 2:**
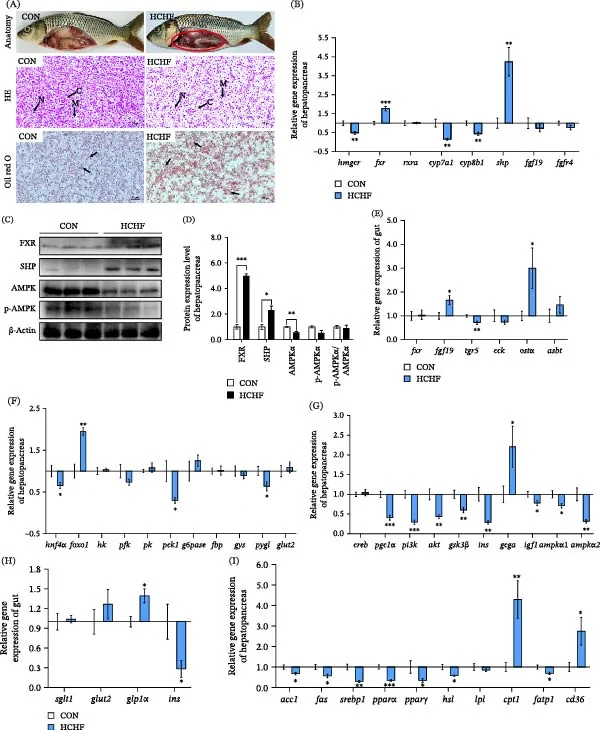
Changes in morphology and gene and protein expression levels of glycolipid metabolism disorder in common carp induced by HCHF diet. (A) Observations of the abdominal anatomy, circled abdominal fat deposits; Hepatopancreas HE staining, 200×, N: cell nucleus; C: cytoplasmic vacuolization; M: cell membranes; Hepatopancreas Oil Red O staining, 200×, arrows in the picture point to fat droplets; (B) Expression of genes related to BA metabolism in the hepatopancreas. (C–D) Expression of proteins and analyses in hepatopancreatic tissue. (E) Expression of genes related to BA metabolism in the gut. (F) Expression of carbohydrate metabolism‐related genes downstream of the FXR/SHP in the hepatopancreas. (G) Expression related to carbohydrate metabolism in the hepatopancreas. (H) Expression related to glycolipid metabolism in the gut. (I) Expression related to lipid metabolism in the hepatopancreas. All the data are presented as the means ± SEM (gene expression, *n* = 8; Western blot, *n* = 3;  ^∗^ *p* < 0.05;  ^∗∗^ *p* < 0.01; and  ^∗∗∗^ *p* < 0.001).

### 3.2. The HCHF Diet Altered the Expression of Metabolism‐Related Genes and Proteins

The FXR/SHP pathway constitutes a vital mechanism in the regulation of BAs and glycolipid metabolism. As shown in Figure 2B, the gene expression levels of hepatopancreatic *fxr* and *shp* in common carp significantly increased (*p* < 0.05) following the consumption of the HCHF diet. Conversely, the expression of downstream genes associated with BA synthesis, namely, *cyp7a1*, and *cyp8b1*, was downregulated (*p* < 0.05) at the mRNA level. Furthermore, the expression of *hmgcr*, a gene implicated in cholesterol synthesis, was significantly reduced (*p* < 0.05). Hepatopancreatic tissues were examined via western blotting on the basis of these findings. FXR and SHP protein expression levels were significantly greater (*p* < 0.05) in the HCHF group than in the CON group, as shown in Figure 2C, D. As for intestinal BA metabolism‐related genes, the HCHF group showed significantly greater *fgf19* mRNA expression, whereas *tgr5* expression was significantly downregulated (*p* < 0.05, Figure 2E). In addition, the mRNA expression of *ostα* was significantly upregulated in the HCHF group (*p* < 0.05, Figure 2E).

Gene expression associated with hepatopancreatic and intestinal carbohydrate metabolism in common carp is impacted by the HCHF diet, as shown in Figure 2F–H. Notably, the mRNA expression of the FXR/SHP downstream genes *hnf4α* and *pck1* was significantly downregulated, whereas *foxo1* mRNA expression was significantly elevated (*p* < 0.05). Furthermore, there was a significant decrease in the expression of genes related to the insulin signaling system, such as *pi3k*, *akt*, and *gsk3β* (*p* < 0.05). Significant downregulation of the mRNA levels of genes linked to insulin secretion (*ins*), *igf1*, and *pygl* (*p* < 0.05) was also detected. Conversely, the expression of the glucagon gene (*gcga*) was upregulated (*p* < 0.05), and *pgc-1α* mRNA levels were significantly decreased (*p* < 0.05). Additionally, the *ampkα1* and *ampkα2* expression levels were significantly lower in the HCHF group than in the CON group, which is consistent with the AMPKα protein expression pattern (*p* < 0.05, Figure 2C, D). However, neither the p‐AMPKα/AMPKα ratio nor the p‐AMPKα level showed any discernible differences (*p* > 0.05, Figure 2C, D). The gut showed significantly elevated expression of the *glp-1* gene and notably reduced expression of the *ins* gene (*p* < 0.05).

Figure 2I depicts the effects of the HCHF diet on the expression of genes involved in the hepatopancreatic lipid metabolism. Compared with those in the CON group, the mRNA expression of key genes involved in hepatopancreatic fatty acid synthesis, *acc1*, *fas*, and the nuclear transcription factor *srebp1*, was significantly lower in the HCHF group (*p* < 0.05). The mRNA expression of the key lipolysis‐related genes *pparα* and *hsl* was significantly reduced (*p* < 0.05); in contrast, *cpt1* mRNA expression was significantly increased (*p* < 0.05). For lipid metabolism transporter genes, *fatp* mRNA expression was significantly downregulated (*p* < 0.05), whereas *cd36* mRNA expression was significantly upregulated (*p* < 0.05).

### 3.3. The HCHF Diet Perturbed the Gut Microbiota and Tissue Functions

We analyzed the gut microbial community of common carp at multiple taxonomic levels to assess the impact of the HCHF diet. The gut phylum was dominated by *Proteobacteria*, *Firmicutes*, *Planctomycetes*, and *Actinobacteria*. Compared with the CON group, the HCHF group presented greater levels of *Proteobacteria*, *bacterium JUN-11*, and *bacterium m5*. In contrast, the abundances of *Firmicutes*, *Planctomycetes*, *Actinobacteria*, *bacterium YC-ZSS-LKJ199*, and *Verrucomicrobia* were lower (Figure 3A). The gut microbiota varied among individuals within the same group at the genus and species levels. At the genus level, the HCHF diet increased the abundance of *Serratia*, *Pseudomonas*, *Psychrobacter*, and *Pantoea* while decreasing the abundance of *Streptococcus*, *Aestuariivirga*, and *Planctomycetaceae bacterium D2* (Figure 3B). At the species level, the abundances of *Serratia liquefaciens*, *Pseudomonas* sp., *Serratia* sp., *Psychorobacter alimentarius*, *Pseudomonas jessenii*, and *Pantoea agglomerans* increased in the HCHF group, whereas the abundances of *Streptococcus iniae*, *Aestuariivirga litoralis*, and *Planctomycetaceae bacterium D2* decreased (Figure 3C).

Figure 3Effects of the HCHF diet on the gut microbiota. (A) Abundance at the phylum level. (B) Abundance at the genus level. (C) Abundance at the species level. (D) Gut histology. GC: goblet cell. A small amount of epithelial cell edema is observed in the mucosal layer of the gut tissue (as indicated by the black arrow within the red circle). (E) Alpha diversity indices. (F) Clustering tree of UPGMA analysis. (G) PCoA. (H–I) Specific gut microbiota analyzed at different taxonomic levels. All the data are presented as the means ± SEM (*n* = 4;  ^∗^ *p* < 0.05;  ^∗∗^ *p* < 0.01; and  ^∗∗∗^ *p* < 0.001).

**Figure:**
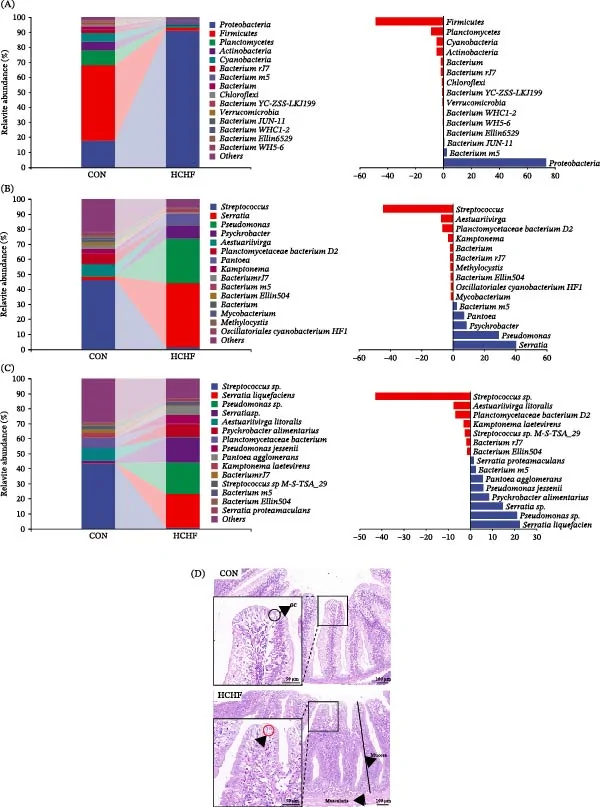

**Figure:**
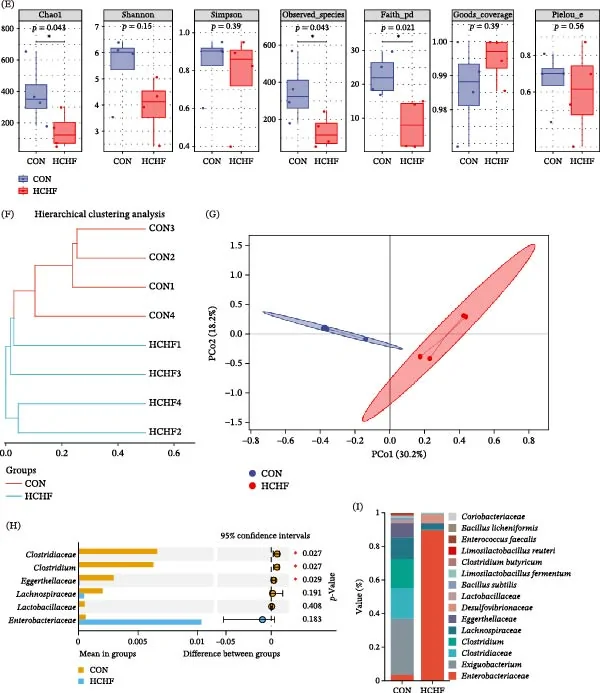

Histological examination of the gut sections revealed that the HCHF group had fewer goblet cells in the intestinal mucosal layer than the CON group did. Concurrently, an increase in connective tissue was observed within the submucosal layer, resulting in the formation of fibrous tissue. Within the microscopic field of sections from the HCHF group, a small amount of epithelial cell edema was observed in the mucosal layer of the gut tissue, accompanied by cellular swelling and rarefied, pale‐stained cytoplasm. (Figure 3D).

Figure 3E shows the diversity. The Chao1 index of the HCHF group was significantly lower than that of the CON group. Similarly, the Shannon and Simpson indices decreased, but there were no notable differences between the two groups. Distance‐based UPGMA and PCoA analyses (Figure 3F, G) clearly revealed separation between the two groups, with PCoA1 and PCoA2 accounting for 30.2% and 18.2% of the variation, respectively. The results of UPGMA were consistent with those of PCoA. The specific gut microbiota was analyzed at different taxonomic levels. At the family level, *Coriobacteriaceae*, *Eggerthellaceae*, *Lachnospiraceae*, and *Lactobacillaceae* were more abundant in the CON group, whereas at the genus level, *Bacillus*, *Clostridium*, and *Exiguobacterium* were enriched in the CON group (Figure 3H, I). At the species level, *Enterococcus faecalis*, BL, *Bacillus subtilis*, *Limosilactobacillus fermentum*, *Limosilactobacillus reuteri*, and *Clostridium butyricum* were increased in the CON group. However, *Enterobacteriaceae* and *Desulfovibrionaceae* were increased in the HCHF group (Figure 3H, I).

### 3.4. Probiotics Ameliorate Metabolic Disorders but Have no Effect on the Growth of Common Carp

Compared with the HCHF group, probiotic supplementation had no significant effect on fish growth, whereas the HSI of common carp was significantly lower in the BL group (*p* < 0.05, Supporting Information Table S6).

In this study, viable counts of the two probiotic strains in feed were monitored during storage at 4°C for 3 days after spray application. Both strains exhibited similar survival patterns. Viable cell counts decreased initially after spraying. Within 24 h, the viable count of LP decreased from 1.74 × 10^8^ CFU g^−1^ to 4.80 × 10^7^ CFU g^−1^, and that of BL decreased from 1.17 × 10^8^ CFU g^−1^ to 3.00 × 10^7^ CFU g^−1^. The two strains stabilized between 24 and 72 h, showing an overall trend of an initial decline followed by a plateau (Figure 4A, B). Throughout storage, viable counts of both probiotics remained within the normal range for probiotic supplementation in feed.

**Figure 4:**
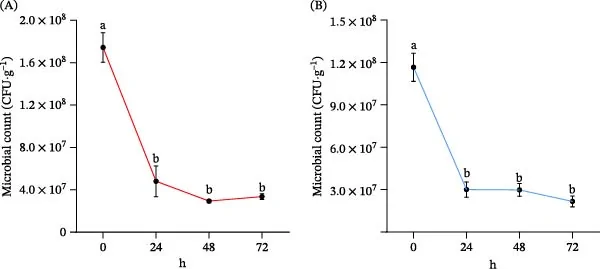
Changes in viable cell counts of *L. plantarum* (A) and *B. licheniformis* (B) in a HCHF diet during storage at 4°C (significance is represented by lowercase letters).

Probiotic supplementation was associated with improved metabolic parameters. Compared with those in the HCHF group, the serum levels of GLU, TG, LDL‐C, T‐CHO, and MDA were significantly lower in the probiotic‐supplemented groups, whereas the activities of the antioxidant enzymes T‐SOD and CAT were greater in these groups (*p* < 0.05). However, the serum AST and ALT levels did not differ significantly among all groups (*p* > 0.05, Figure 5A–I). Compared with those in the HCHF group, the hepatopancreas HDL‐C level in the BL group was significantly greater, and the TBA content in the LP group was significantly lower (*p* < 0.05, Figure 5J, M). The ALT level was significantly downregulated in all probiotic‐supplemented groups, and the activity of the antioxidant enzyme T‐SOD was increased in the BL group (*p* < 0.05, Figure 5N, O). No significant differences were observed in hepatopancreas T‐CHO and AST levels (*p* > 0.05, Figure 5L, P). Compared with those in the HCHF group, the levels of TBA and LD in the gut of the BL group were significantly greater (*p* < 0.05, Figure 5Q, R).

**Figure 5:**
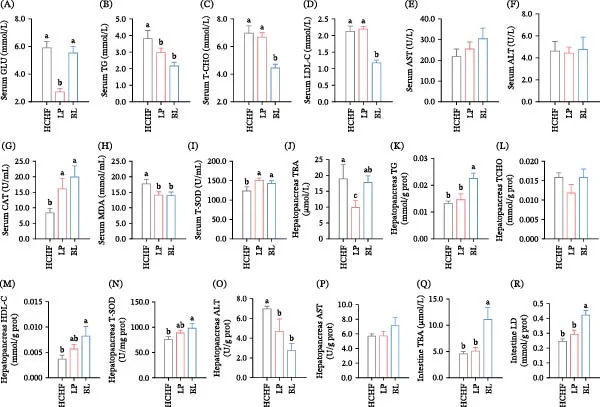
Analysis of the ability of *L. plantarum* and *B. licheniformis* to improve metabolic disorders. (A) Serum Glu. (B) Serum TG. (C) Serum T‐CHO. (D) LDL‐C (E) Serum AST. (F) Serum ALT. (G) Serum CAT. (H) Serum MDA. (I) Serum T‐SOD. (J) Hepatopancreatic TBA. (K) Hepatopancreatic TG. (L) Hepatopancreatic T‐CHO. (M) Hepatopancreas HDL‐C. (N) Hepatopancreatic T‐SOD. (O) Hepatopancreatic ALT. (P) Hepatopancreatic AST. (Q) Gut TBA. (R) Gut LD. All the data are presented as the means ± SEM (*n* = 8; significance is represented by lowercase letters).

### 3.5. Probiotics Could Modulate BA Metabolic Profiles

Figure 6A shows the expression of BA metabolism‐related genes. In the hepatopancreas, compared with that in the HCHF group, the mRNA expression level of *hmgcr* was significantly lower in the BL group (*p* < 0.05), the mRNA expression level of *fxr* was significantly greater (*p* < 0.05), and the mRNA expression of *shp*, a downstream gene of FXR, was significantly greater (*p* < 0.05). In the BL group, the mRNA expression of *cyp7a1* was significantly downregulated. Additionally, the mRNA expression of *cyp8b1* was significantly downregulated in both the LP and the BL groups (*p* < 0.05). The mRNA expression level of *fgf19* was significantly decreased in both the LP and the BL groups, whereas in the LP group, the mRNA expression level of *fgfr4* (the receptor for FGF19) was significantly increased (*p* < 0.05).

Figure 6Analysis of the ability of *L. plantarum* and *B. licheniformis* to regulate bile acid metabolism. (A) Expression of genes related to BA metabolism in the hepatopancreas. (B–D) Proportions and contents of bile acids at different classification levels. (E) Statistical analysis of bile acid categories with significant changes. (F) Correlation analysis between significantly changed bile acid and serum and hepatopancreas biochemical indicators. All the data are presented as the means ± SEM (gene expression, *n* = 8; bile acid metabolic profile and correlation analysis, *n* = 6; significance is represented by lowercase letters.  ^∗^ *p* < 0.05;  ^∗∗^ *p* < 0.01; and  ^∗∗∗^ *p* < 0.001).

**Figure:**
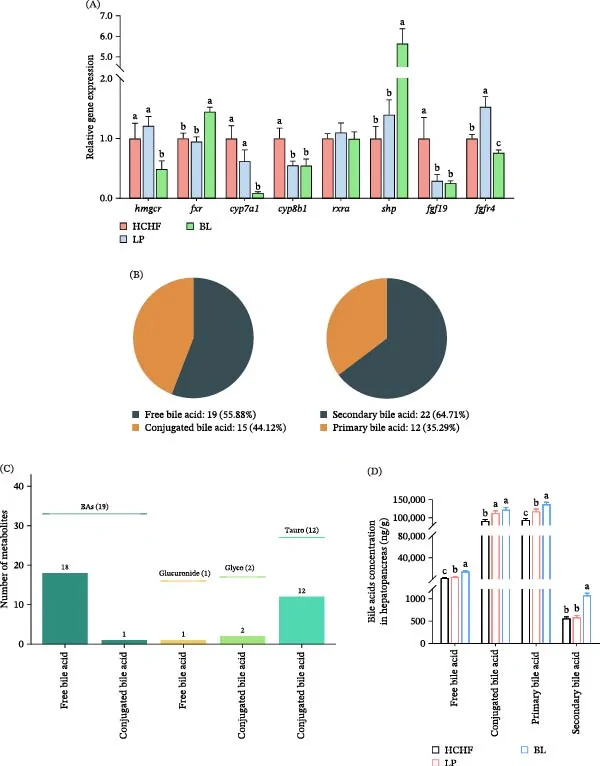

**Figure:**
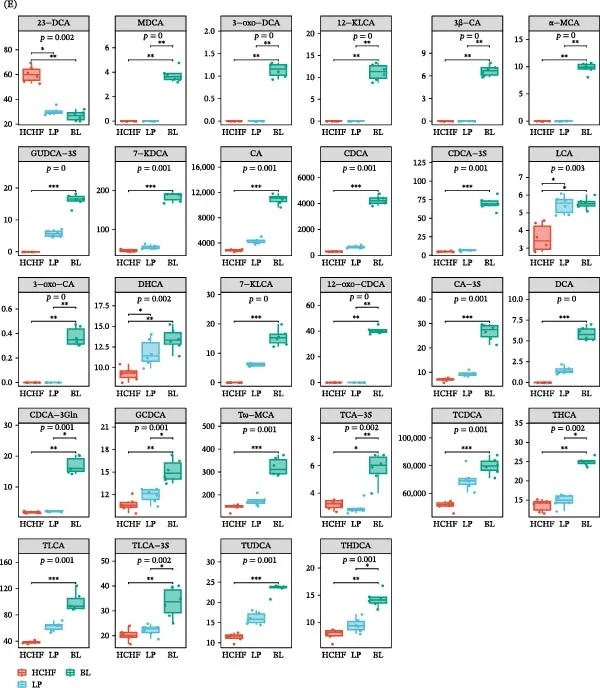

**Figure:**
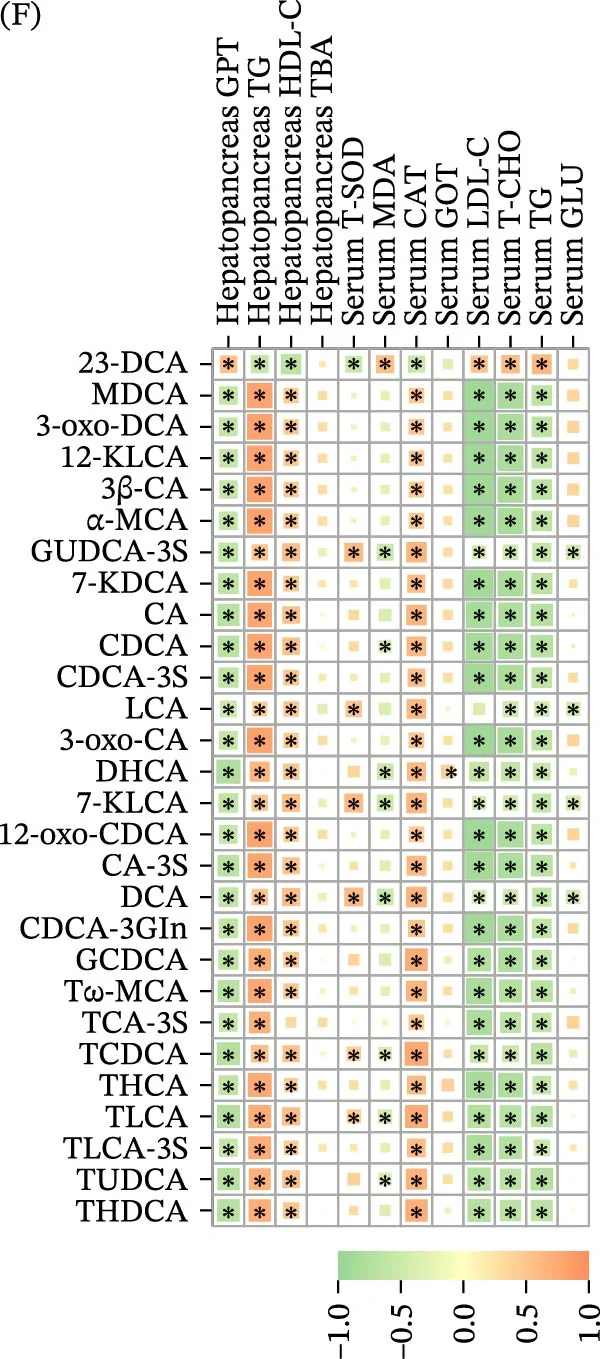

Figure 6B–D shows the proportions and concentrations of BAs at different classification levels. In general, free BAs and secondary BAs accounted for a relatively high proportion of the TBAs in the hepatopancreas. In terms of structure, free BAs and taurine‐conjugated BAs were the most abundant in the hepatopancreas. Specifically, both free and conjugated BAs, as well as secondary and primary BAs, were increased in the probiotic‐supplemented groups.

Statistical analysis of BAs with significant alterations in the hepatopancreas revealed that most BAs (e.g., CA, CDCA, LCA, DCA, and DHCA) were significantly increased in the probiotic‐supplemented groups, with more pronounced changes observed in the BL group than in the LP group (*p* < 0.05, Figure 6E and Supporting Information Table S7). Only 23‐DCA was significantly decreased in the probiotic‐supplemented groups (*p* < 0.05, Figure 6E and Supporting Information Table S7).

Correlation analysis (Figure 6F) was performed on the serum, hepatopancreatic biochemical indices, and BA expression profiles, and the results demonstrated that most BAs were negatively correlated with the serum TG, T‐CHO, LDL‐C, and hepatopancreatic ALT levels but positively correlated with the serum CAT, hepatopancreatic HDL‐C, and TG levels.

### 3.6. Probiotics Modulated Intestinal BA Metabolism and Hepatopancreatic Metabolism‐Related Gene Expression

Compared with those in the HCHF group, the mRNA expression levels of *fgf19* in the gut were significantly lower in all probiotic‐supplemented groups (*p* < 0.05, Figure 7A), and the mRNA expression of *tgr5* was significantly lower in the BL group (*p* < 0.05, Figure 7A). Additionally, *ostα* mRNA expression was significantly downregulated in both the LP and BL groups (*p* < 0.05, Figure 7A), while *asbt* expression showed no significant change (*p* > 0.05, Figure 7A).

**Figure 7:**
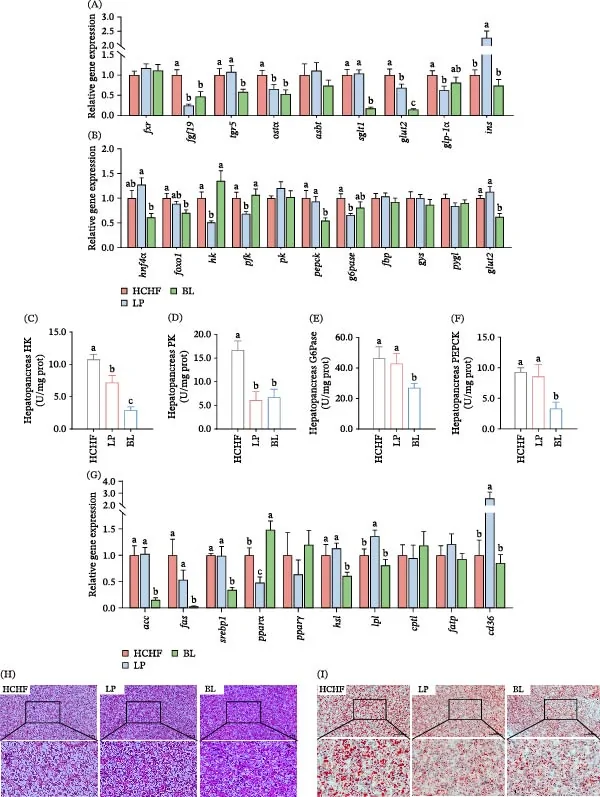
Effects of *L. plantarum* and *B. licheniformis* on the expression of metabolism‐related genes and morphology. (A) Expression of genes related to BA metabolism in the gut. (B) Expression of carbohydrate metabolism‐related genes downstream of the FXR/SHP in the hepatopancreas. (C–F) Hepatopancreatic carbohydrate metabolism enzymes: HK, PK, G6Pase, and PEPCK. (G) Expression related to lipid metabolism in the hepatopancreas. (H) Hepatopancreas H&E staining of the hepatopancreas, 200×. (I) Hepatopancreas Oil Red O staining, 200×. All the data are presented as the means ± SEM (*n* = 8; significance is represented by lowercase letters).

The expression of Glu metabolism‐related genes is shown in Figure 7A, B. Compared with those in the HCHF group, the mRNA expression levels of *hnf4α* and *foxo1*, target genes of FXR/SHP, were significantly lower in the BL group (*p* < 0.05). The results for the expression of key glycolytic enzyme‐encoding genes revealed that the mRNA expression levels of *hk* and *pfk* were significantly downregulated in the LP group (*p* < 0.05). For key gluconeogenic enzyme‐encoding genes, downstream targets of HNF4α and FOXO1, the mRNA expression of *pepck* was suppressed in the BL group, whereas the mRNA expression of *g6pase* was suppressed in the LP group (*p* < 0.05). With respect to Glu transporter genes, the mRNA expression of *glut2* was significantly downregulated in the BL group (*p* < 0.05). In the gut, probiotics significantly downregulated the mRNA expression of the Glu transporter genes *sglt1* and *glut2*, as well as *glp-1α* (*p* < 0.05, Figure 7A). In the LP group, the mRNA expression of *ins* was significantly increased (*p* < 0.05, Figure 7A).

The activities of Glu metabolic enzymes in the hepatopancreas are shown in Figure 7C–7F. Compared with those in the HCHF group, the activities of the key glycolytic enzymes HK and PK were significantly lower in the probiotic‐supplemented groups (*p* < 0.05). In the BL group, the activities of the key gluconeogenic enzymes PEPCK and G6Pase were significantly decreased (*p* < 0.05).

The expression of lipid metabolism‐related genes is shown in Figure 7G. Compared with those in the HCHF group, the mRNA expression levels of the lipid synthase genes *fas*, *acc1*, and *srebp1* in the hepatopancreas of the BL group were significantly lower (*p* < 0.05); the mRNA expression level of the lipid catabolic gene *hsl* in the BL group was significantly lower, whereas that of *lpl* in the LP group was significantly greater (*p* < 0.05). Additionally, the mRNA expression level of the lipid transport‐related gene *cd36* in the LP group was significantly increased (*p* < 0.05).

Probiotics could ameliorate HCHF diet‐induced hepatocellular vacuolization and alleviate lipid deposition in the hepatopancreas (Figure 7H, I). The results of Oil Red O staining demonstrated that lipid droplets were extensively accumulated with large morphologies in the HCHF group, whereas in the probiotic‐supplemented groups, the lipid droplets were reduced in both size and number.

### 3.7. Effects of Probiotics on the Composition and Function of the Gut Microbiota

Probiotics induced alterations in the gut microbiota of common carp (Figure 8A–F). *Proteobacteria* was the predominant gut phylum across all groups, whereas the relative abundances of *Fusobacteriota*, *Firmicutes*, *Actinobacteriota*, *Verrucomicrobiota*, and *Bacteroidota* were relatively low. Statistical analyses revealed that, at the phylum level, compared with those in the HCHF group, the relative abundances of *Fusobacteriota* and *Firmicutes* increased, whereas that of *Proteobacteria* decreased in both the LP and BL groups. At the genus level, the relative abundance of *Cetobacterium* was significantly elevated in both the LP and BL groups, whereas the abundance of *Pseudomonas* was reduced in the LP group alone. At the species level, the relative abundance of ASVs classified as *Cetobacterium somerae* significantly increased in both the LP and BL groups compared with the HCHF group, and the abundance of *Pseudomonas* species decreased in the LP group.

Figure 8Effects of *L. plantarum* and *B. licheniformis* on the gut microbiota. (A–B) Abundance and statistical analysis at the phylum level. (C–D) Abundance and statistical analysis at the genus level. (E–F) Abundance and statistical analysis at the species level. (G) Alpha diversity indices. (H) PCoA. (I) Clustering tree of UPGMA analysis. All the data are presented as the means ± SEM (*n* = 8; significance is represented by lowercase letters).

**Figure:**
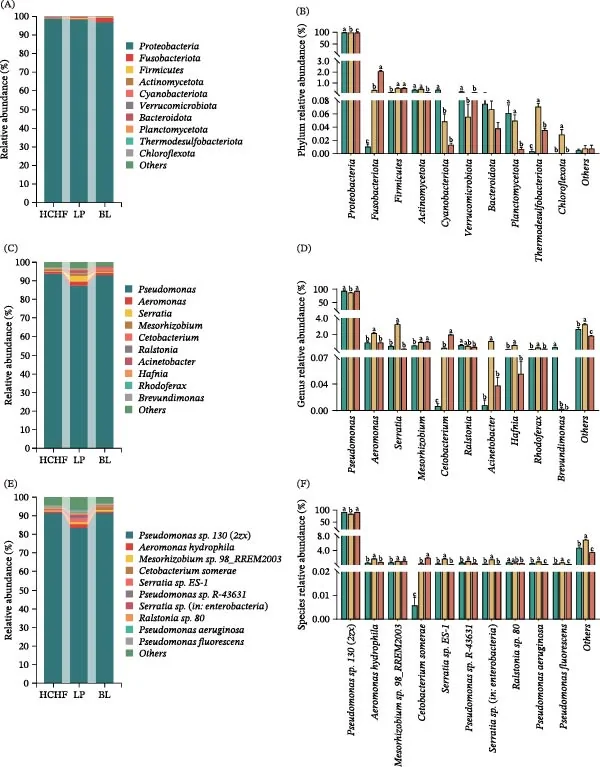

**Figure:**
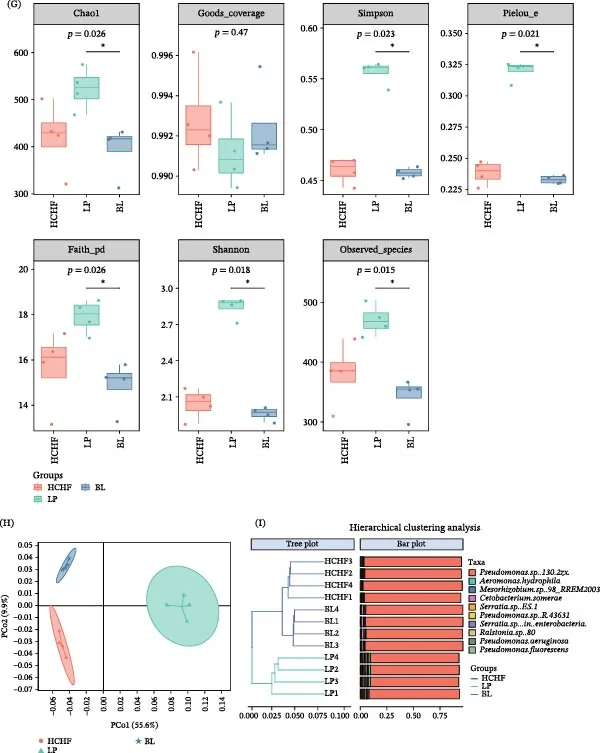

Alpha‐diversity indices reflect the richness of a species within an ecosystem, whereas beta‐diversity indices are used to evaluate the diversity among different microbial communities. Compared with those in the HCHF group, the Chao 1, Shannon, and Simpson indices in the LP group tended to increase, but the difference was not statistically significant. The results of PCoA and UPGMA revealed sample segregation between the HCHF group and the LP/BL groups, indicating distinct differences in the gut microbial community structure among these groups (Figure 8G–I).

Taxonomic levels of the gut microbiota and Venn diagram analysis revealed a total of 50 shared ASVs across all groups (Figure 9A, B). At the genus level, the dominant gut microbes of each group are presented in Figure 9C; the genera *Clostridium* (LP group) and *Cetobacterium* and *Bacillus* (BL group) presented relatively high abundances. OPLS‐DA demonstrated that the HCHF group and probiotic‐supplemented groups presented significant differences in the gut microbiota composition (Figure 9D). Cladogram and LEfSe analyses (LDA > 3.16) were performed to identify the biomarker species for each group. The results revealed that the microbial taxa enriched in the HCHF group included *Enterobacteriaceae*, *Prevotellaceae*, etc.; the LP group was enriched with *Moraxellaceae*, *Comamonadaceae*, *Rhodoferax fermentans*, etc.; and the BL group was enriched with *C. somerae*, etc. (Figure 9E, F).

Figure 9Effects of *L. plantarum* and *B. licheniformis* on specific intestinal species of the gut microbiota. (A) Statistics of the number of taxonomic units. (B) Venn diagram. (C) Heatmap of the species composition. (D) OPLS‐DA. (E–F) Cladogram and LDA effect size analyses (threshold ≥ 3.16).

**Figure:**
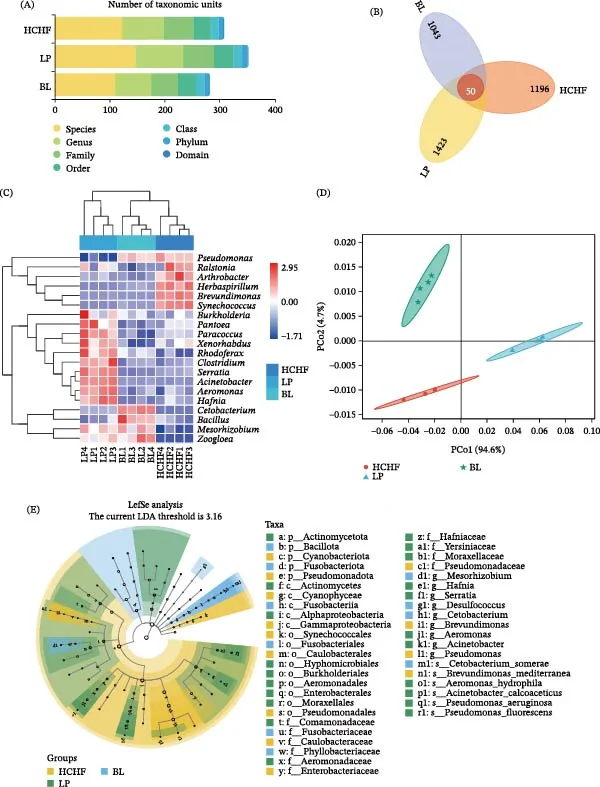

**Figure:**
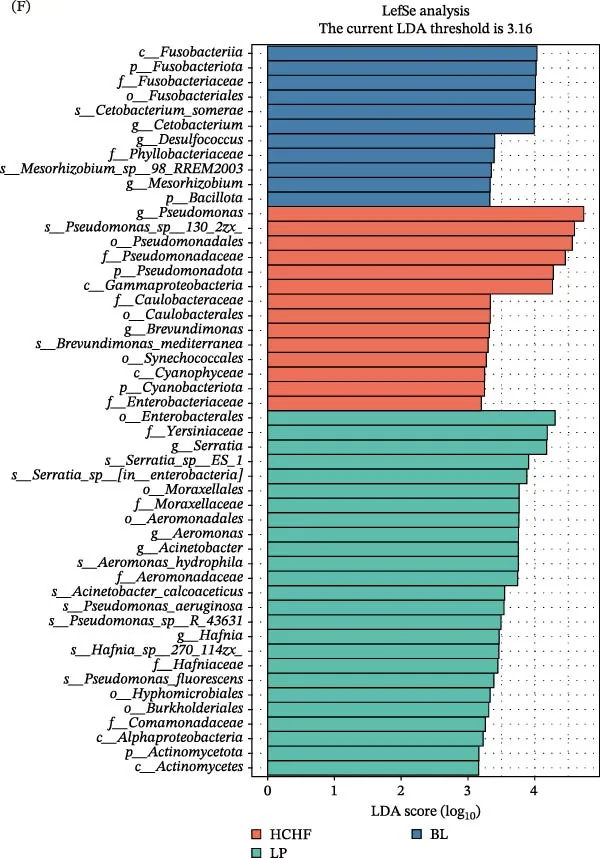

To assess how the administered probiotics influenced the host, we further examined the abundance of specific gut microbes. Compared with those in the HCHF group, *C. somerae* exhibited the most significant enrichment in the gut in both the LP and the BL groups (*p* < 0.05, Figure 10A, B). At the genus level, we subsequently analyzed the gut microbial genera with potential BSH activity and found that the abundance of *Clostridium* significantly increased in the LP group, whereas that of *Bacillus* significantly increased in the BL group (*p* < 0.05, Figure 10C, D).

Figure 10Effects of *L. plantarum* and *B. licheniformis* on the functions of the gut microbiota. (A–B) Statistical analysis of specific microorganisms in the gut revealed that the abundance of *Cetobacterium somerae* in the probiotic treatment group increased. (C–D) Statistical analysis of potentially BSH‐active taxa revealed that the abundance of *Clostridium* was greater in the LP group, whereas the abundance of *Bacillus* was greater in the BL group. (E) Correlation analysis between the gut microbiota and serum and hepatopancreas biochemical indicators. (F) Correlation analysis between the gut microbiota and bile acid profile. (G) Functional unit PCoA. (H) Metabolic pathway statistics.

**Figure:**
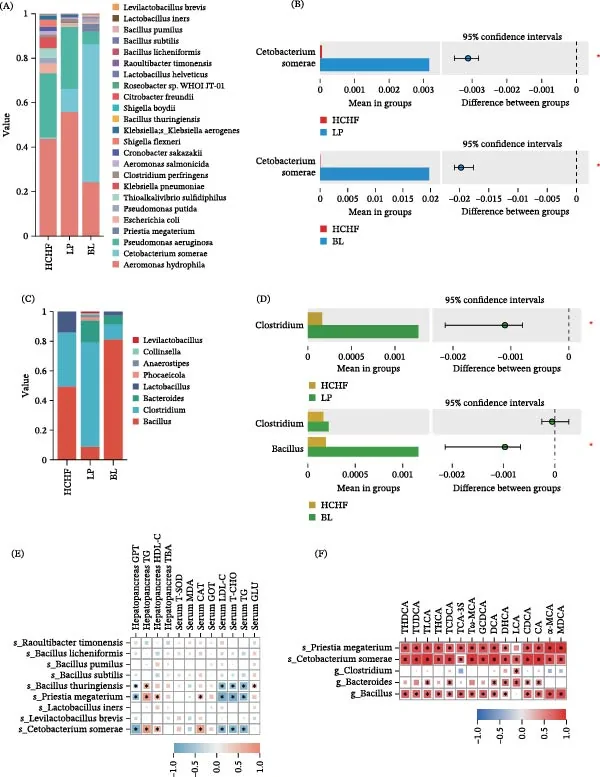

**Figure:**
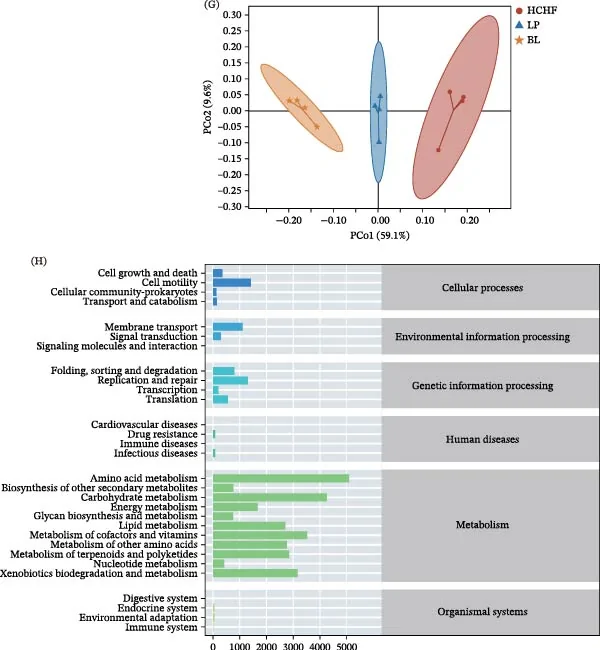

The present study performed correlation analyses between the gut microbiota and serum/hepatopancreatic biochemical indices as well as between the gut microbiota and BA profiles. The results demonstrated that *Bacillus thuringiensis*, *Priestia megaterium*, and *C. somerae* were negatively correlated with serum TG, T‐CHO, LDL‐C, and hepatopancreatic ALT (Figure 10E). Additionally, *Priestia megaterium*, *C. somerae*, *Bacteroides*, and *Bacillus* were positively correlated with BA (Figure 10F).

Finally, the potential functions of the gut microbiota were investigated via PCoA and KEGG pathway analyses. The results revealed that intestinal microbes had prominent effects on cellular processes, environmental information processing, genetic information processing, and metabolism at the primary pathway level. The present study focused primarily on metabolic functional pathways, and the findings demonstrated that intestinal microbes play a dominant role in multiple metabolic processes, including amino acid metabolism, carbohydrate metabolism, metabolism of cofactor and vitamin, xenobiotics biodegradation and metabolism, lipid metabolism, metabolism of other amino acid, metabolism of terpenoids and polyketide, energy metabolism, and biosynthesis of other secondary metabolites (Figure 10G, H).

### 3.8. Effects of Probiotics on Gut Metabolites

The chemical classification and statistical attribution of these metabolites in the intestinal contents of common carp are illustrated in Figure 11A, B. The compounds, organic acids and derivatives, lipids and lipid‐like molecules, and benzenoids presented the highest relative abundances. Additionally, the identified metabolite categories included alkaloids, amino acids and peptides, fatty acids, carbohydrates, organic oxygen compounds, and organic nitrogen compounds, among others.

Figure 11Effects of *L. plantarum* and *B. licheniformis* on intestinal metabolites. (A–B) Classification proportions of metabolites in positive ion and negative ion modes. (C) Sample correlation analysis. (D–E) Univariate statistical analysis of metabolites detected in positive and negative ion modes in the *L. plantarum* treatment group. (F–G) Univariate statistical analysis of metabolites detected in positive and negative ion modes in the *B. licheniformis* treatment group. (H–I) Multivariate OPLS‐DA analysis of metabolites detected in positive and negative modes in the *L. plantarum* treatment group. (J–K) Multivariate OPLS‐DA analysis of metabolites detected in positive and negative modes in the *B. licheniformis* treatment group. (L) Statistics of the differentially abundant metabolites in the LP group compared with those in the HCHF group. (M) Statistics of differentially abundant metabolites in the BL group compared with those in the HCHF group.

**Figure:**
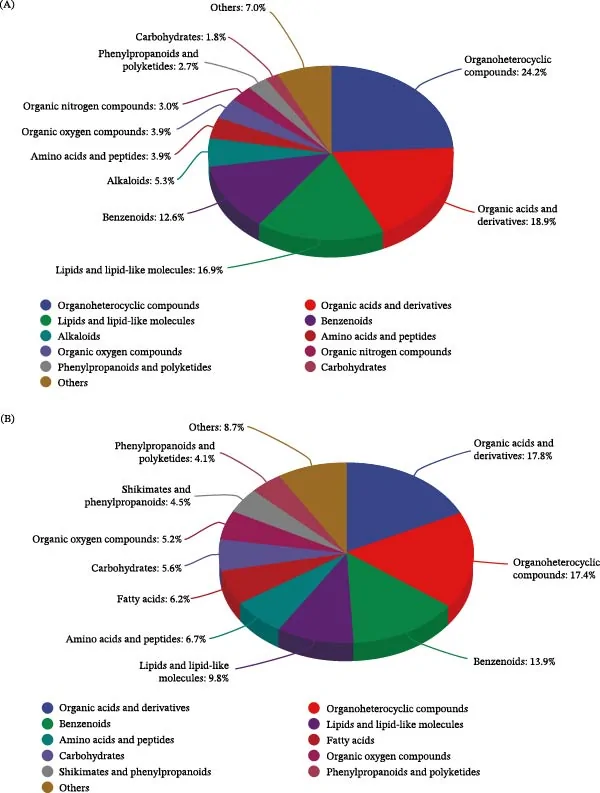

**Figure:**
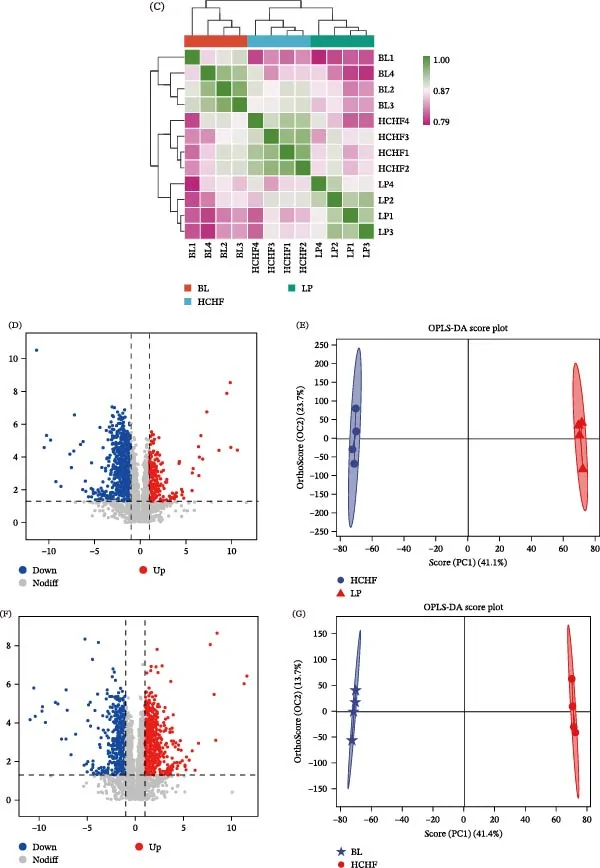

**Figure:**
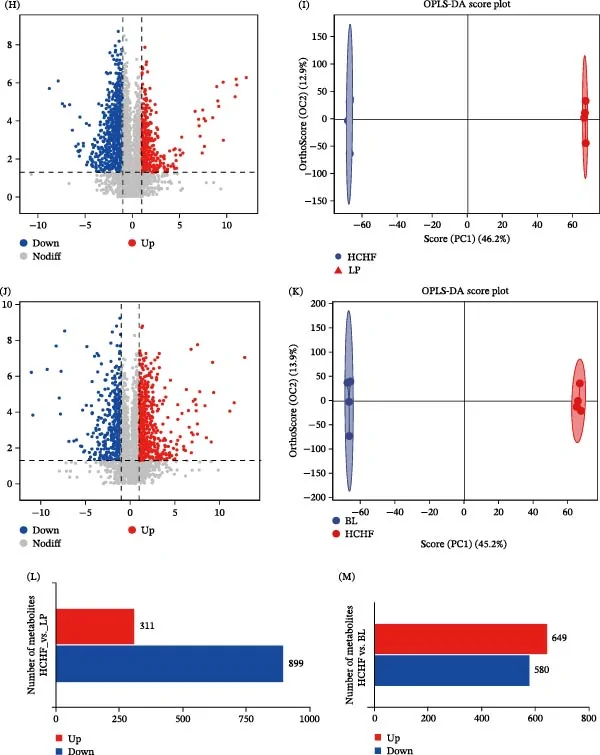

The present study subsequently screened for significantly differentially expressed metabolites. Prior to this step, the correlation between metabolite expression levels among samples was examined. The results demonstrated that the correlation of metabolite expression levels among the samples in this study met the experimental requirements, indicating the high reliability and reproducibility of the experiment (Figure 11C). Volcano plots and OPLS‐DA were employed to identify the significantly differentially abundant metabolites between the HCHF group and the LP group, as well as between the HCHF group and the BL group, in both positive and negative ion modes. Compared with those in the HCHF group, a total of 311 metabolites were significantly upregulated and 899 were significantly downregulated in the LP group; in the BL group, 649 metabolites were significantly upregulated and 580 were significantly downregulated (Figure 11D–M).

The results of the KEGG pathway enrichment analysis revealed that these differentially abundant metabolites were significantly enriched in the following pathways in the LP group: sphingolipid metabolism; glycerophospholipid metabolism; apoptosis; autophagy‐animal; purine metabolism; necroptosis; linoleic acid metabolism; fructose and mannose metabolism; amino sugar and nucleotide sugar metabolism; carbon metabolism; pyruvate metabolism; biosynthesis of nucleotide sugars; oxidative phosphorylation; citrate cycle (TCA cycle); glyoxylate and dicarboxylate metabolism; ABC transporters; biosynthesis of cofactors; ascorbate and aldarate metabolism; pentose and glucuronate interconversions; metabolic pathways; alanine, aspartate, and glutamate metabolism; glycosylphosphatidylinositol (GPI)‐anchor biosynthesis; lysosome, and phenylalanine, tyrosine, and tryptophan biosynthesis (Figure 12A, B).

**Figure 12:**
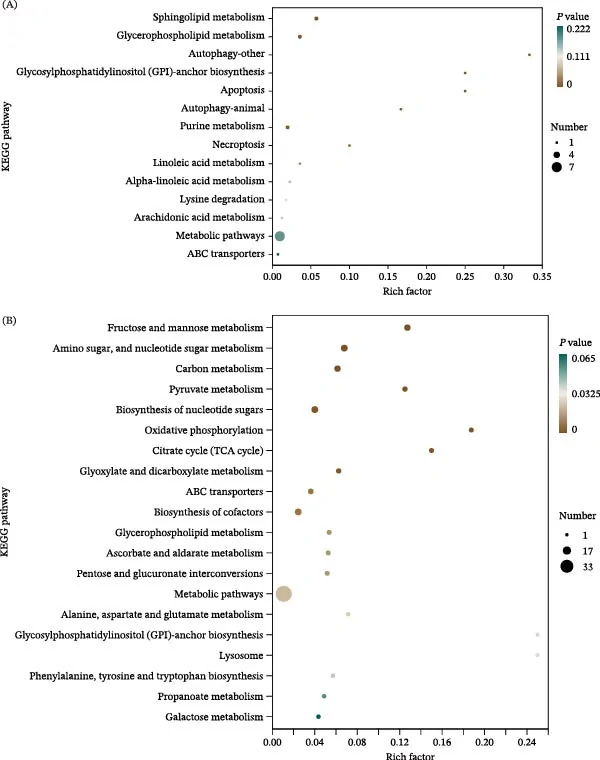
(A) KEGG signaling pathways significantly upregulated in the LP group under positive ion mode. (B) KEGG signaling pathways significantly upregulated in the LP group under negative ion mode.

Similarly, in the BL group, these differentially abundant metabolites were significantly enriched in the following pathways: glycerophospholipid metabolism; autophagy‐animal; GPI‐anchor biosynthesis; mTOR signaling pathway; FoxO signaling pathway; regulation of actin cytoskeleton; ABC transporters; nicotinate and nicotinamide metabolism; endocytosis; cysteine and methionine metabolism; necroptosis; amino sugar and nucleotide sugar metabolism; fructose and mannose metabolism; biosynthesis of cofactors; phenylalanine, tyrosine and tryptophan biosynthesis; biosynthesis of nucleotide sugars; biosynthesis of amino acids; pyruvate metabolism; lysosome; purine metabolism; citrate cycle (TCA cycle); metabolic pathways; ascorbate and aldarate metabolism; 2‐oxocarboxylic acid metabolism; carbon metabolism; propanoate metabolism; oxidative phosphorylation; tryptophan metabolism; galactose metabolism; phenylalanine metabolism; nicotinate and nicotinamide metabolism; lysine degradation; pentose and glucuronate interconversions; taurine and hypotaurine metabolism; glyoxylate and dicarboxylate metabolism; alanine, aspartate and glutamate metabolism; and arginine and proline metabolism (Figure 13A, B).

**Figure 13:**
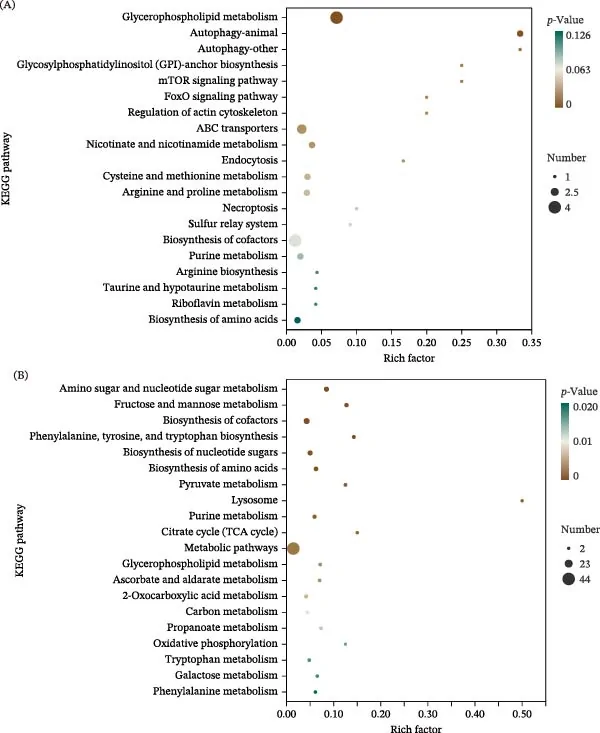
(A) KEGG signaling was significantly upregulated in the BL group in positive mode. (B) KEGG signaling pathway was significantly upregulated in the BL group in negative mode.

In this study, metabolites in the significantly upregulated and enriched KEGG pathways were identified. Under both positive and negative ion modes, a total of 43 significantly upregulated differentially abundant metabolites were identified in the LP group (Supporting Information Table S8), along with 60 significantly upregulated differentially abundant metabolites in the BL group (Supporting Information Table S9). Correlation analysis was subsequently performed between the screened differentially abundant metabolites and the intestinal probiotics identified in the above study. The results revealed that in the LP group, *C. somerae* was positively correlated with the vast majority of these metabolites, including M307 (sphingosine), M2252 (PE (22:4(7Z,10Z,13Z,16Z)/22:2(13Z,16Z))), M110 (phosphoglycolic acid), M245 (fumaric acid), M381 (malic acid), M488 (succinate), M435 (mesaconic acid), M436 (itaconic acid), etc. (Figure 14A). In the BL group, *Priestia megaterium* and *C. somerae* were positively correlated with the majority of the metabolites, including M10 (nicotinamide), M100 (creatinine), M172 (SM(d18:1/18:0)), M2118 (PC(20:4(8Z,11Z,14Z,17Z)/14:1(9Z))), M266 (acetylcholine), M63 (riboflavin), M88 (betaine), M116 (sn‐glycerol 3‐phosphate), M124 (quinic acid), M20 (tryptophan), M285 (pyruvate), M342 (mevalonic acid), M381 (malic acid), M4 (taurine), M488 (succinate), M62 (cholic acid), etc. (Figure 14B).

**Figure 14:**
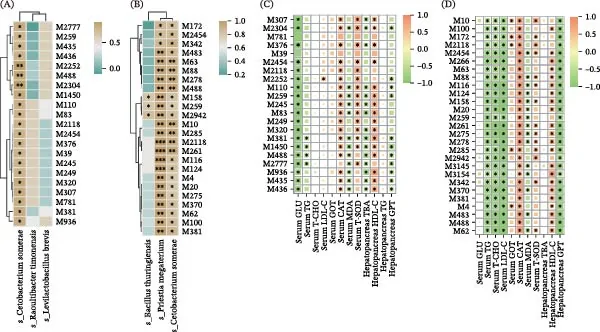
Correlation analysis. (A) Correlation analysis between the gut microbiota and differentially abundant metabolites in the LP group. (B) Correlation analysis between the gut microbiota and differentially abundant metabolites in the BL group. (C) Correlation analysis between serum and hepatopancreas biochemical indices and differentially abundant metabolites in the *L. plantarum* treatment group. (D) Correlation analysis between serum and hepatopancreas biochemical indices and differentially abundant metabolites in the *B. licheniformis* treatment group.

Finally, a correlation analysis was performed between the significantly altered metabolites and serum and hepatopancreatic biochemical indices. The results revealed that in the LP group, serum GLU, MDA, and other related indices were negatively correlated with the majority of metabolites, whereas serum CAT, T‐SOD, and hepatopancreatic HDL‐C were positively correlated with the majority of metabolites (Figure 14C); additionally, serum TG, T‐CHO, LDL‐C, and other related indices in the BL group were negatively correlated with the majority of metabolites, whereas serum CAT, T‐SOD, and hepatopancreatic HDL‐C were positively correlated with the majority of metabolites (Figure 14D).

## 4. Discussion

In humans, excess dietary fat is more likely to promote adipose tissue accumulation than an equivalent excess of carbohydrate. Furthermore, the consumption of surplus carbohydrates elevates carbohydrate oxidation levels and energizes metabolic processes, consequently resulting in the retention of 75%–85% of any excess energy. In contrast, a high intake of fat diminishes energy expenditure within the body, prompting the retention of 90%–95% of surplus energy [35]. Additionally, when fed diets with varying carbohydrate‒lipid ratios, grass carp moderately utilized both dietary fat and carbohydrates. However, their capacity to use fat is slightly greater than that of carbohydrates [36]. In the present study, the final body weight, weight gain rate, specific growth rate, and hepatosomatic index of common carp significantly increased following the consumption of the HCHF diet. Consequently, there was a significant increase in crude fat levels in the whole fish as well as in the tissues of the muscles and hepatopancreas. These findings indicate that the HCHF diet is associated with enhanced growth in common carp.

The effects of probiotics on growth vary across studies. Studies have shown that the synergistic application of BL and *Bacillus subtilis* can attenuate body weight gain in C57BL/6J mice fed a high‐fat diet [37]. Other studies have demonstrated that diabetic mice treated with *Lactobacillus paracasei* NL41 exhibit reduced free fatty acid levels and increased body weight gain [38]. In fish, several probiotics have been shown to exert growth‐promoting effects. For example, dietary supplementation with *Lactobacillus rhamnosus* JCM5805 can enhance the growth performance and feed utilization efficiency of Nile tilapia [39, 40]. Similarly, dietary supplementation with *Bacillus amyloliquefaciens* SS1 can promote growth and improve the feed utilization efficiency of Nile tilapia [41]. In the present study, LP and BL had no significant effects on the growth performance of common carp fed the HCHF diet. This result may reflect the fact that the growth of common carp is regulated primarily by dietary nutritional components, whereas probiotics alleviate only hepatic and intestinal damage without resulting in improved nutrient digestion, absorption, and utilization.

BA metabolism is governed by a negative feedback mechanism and dietary factors. Upon food intake, the gallbladder secretes large amounts of BAs into the gut under the stimulation of cholecystokinin, facilitating lipid breakdown. Moreover, suppressed expression of FXR/SHP activates the transcription of CYP7A1 and CYP8B1, thereby promoting BA synthesis. Conversely, elevated BA levels inhibit hepatic synthesis by activating hepatic FXR/SHP expression, which suppresses the transcription of BA synthesis‐related genes (CYP7A1 and CYP8B1) and reduces BA production in the liver. Additionally, intestinal FXR enhances the FGF15/19 expression. Once transported to the liver via the bloodstream, FGF15/19 bind to FGFR4, triggering an extracellular signal‐regulated protein kinase (ERK1/2) cascade. This cascade inhibits CYP7A1 gene transcription and curtails BA synthesis [19, 42, 43]. Studies have shown that a carbohydrate‐rich diet increases BA production in humans [44], whereas diets high in fat increase serum BA levels in people with lipid metabolism disorders [45]. In the model‐building trial, HCHF feeding significantly upregulated hepatic FXR and SHP expression while suppressing *cyp7a1* and *cyp8b1* transcription, which is consistent with the classic negative feedback regulation of BA synthesis. Nevertheless, the hepatic TBA content was elevated in the HCHF group [46]. This apparent paradox reflects the fact that the hepatic BA content is determined by the net balance between synthesis, intestinal reabsorption, biliary excretion, and uptake from circulation, rather than by synthesis alone. Several factors likely contribute to this phenomenon in the context of HCHF feeding. First, the high‐fat content of the diet stimulates cholecystokinin secretion and gallbladder contraction, which increases the enterohepatic recycling flux of BAs independently of de novo synthesis [47]. Second, HCHF‐induced hepatopancreatic damage, as evidenced by elevated transaminase activities, hepatocyte vacuolization, and disorganized cellular architecture, may impair biliary excretion and lead to intrahepatic BA retention [48]. Third, the negative feedback regulation of BA synthesis via the FXR/SHP pathway requires a certain time lag to fully suppress *cyp7a1* transcription [49, 50]. The sampling time point in this study may have captured an intermediate state in which synthesis was partially reduced but still contributed to the hepatic pool. These mechanisms indicate that the elevated hepatic BA pool in the HCHF group reflects enhanced enterohepatic recycling and potential excretory impairment rather than a failure of FXR‐mediated feedback repression.

Probiotic supplementation further altered BA dynamics in the intervention trial. Compared with the HCHF group, LP and BL significantly elevated specific BA species in the hepatopancreas despite downregulating *cyp7a1* and *cyp8b1*. This paradox likely reflects enhanced intestinal reabsorption driven by changes in the gut microbiota. Probiotics‐enriched taxa with potential bile salt hydrolase activity, including *Clostridium* and *Bacillus*, which deconjugate conjugated BAs into unconjugated forms [51, 52]. These unconjugated forms are more readily absorbed via passive diffusion, increasing their return to the liver and expanding the hepatic pool. Two observations support this interpretation: elevated intestinal TBA content in the BL group and downregulation of intestinal *ostα* expression in both probiotic groups. The latter likely represents a compensatory response that limits basolateral efflux. Thus, the expanded hepatic BA pool in probiotic groups probably results from enhanced reabsorption of deconjugated BAs, on top of the HCHF‐induced alterations already discussed.

An apparent discrepancy exists between the decreased intestinal *fgf19* expression and the sustained repression of hepatic *cyp7a1* in the probiotic groups. Canonically, FGF19 from the intestine binds hepatic FGFR4 to suppress CYP7A1 transcription, so reduced *fgf19* would be expected to relieve *cyp7a1* repression. However, CYP7A1 is coregulated by both the endocrine FGF19/FGFR4 pathway and the intrahepatic FXR/SHP axis, which is activated by local BA ligands [19]. In our model, the expanded BA pool serves as a source of potent endogenous FXR agonists, directly activating intrahepatic FXR/SHP and overriding the reduced endocrine FGF19 input to maintain *cyp7a1* repression. Thus, the local FXR/SHP signaling, driven by the enlarged BA pool, prevails over the diminished FGF19 signal. Notably, BL exerted a more pronounced regulatory effect on BA profiles than that of LP, consistent with its stronger activation of hepatic FXR/SHP expression. Nevertheless, it should be acknowledged that the current BA analysis was primarily focused on the hepatopancreas, with only the TBA content measured in the intestinal lumen. Profiling of individual BA species in the intestinal contents would provide more direct evidence for the altered enterohepatic circulation. Future studies incorporating comprehensive intestinal luminal BA metabolomics, combined with fecal excretion analysis, will be necessary to fully characterize the BA flux along the gut–liver axis.

The primary sources of intracellular cholesterol are its synthesis within organisms and its uptake from outside sources. A diet rich in carbohydrates and fats can increase serum cholesterol and LDL‐C levels, leading to lipid abnormalities. The cholesterol consumed enters the bloodstream and is transported by LDL‐C, which carries cholesterol from the liver to various body tissues. It enters cells through endocytosis, facilitated by LDL‐C receptors on the cell membrane. Conversely, HDL‐C removes cholesterol from tissues back to the liver for metabolism. When intracellular cholesterol levels increase excessively, the replenishment of LDL‐C receptors decreases, consequently reducing blood cholesterol absorption. The crucial enzyme in cholesterol synthesis is *hmgcr*, which governs de novo cholesterol synthesis. Cholesterol can inhibit HMGCR activity, thereby diminishing cholesterol synthesis in the liver. This study revealed that the T‐CHO, LDL‐C, and HDL‐C levels in the serum were elevated; the LDL‐C level in the hepatopancreas was decreased; the expression of *hmgcr* was decreased; and the TBA level was increased in common carp fed the HCHF diet. We speculated that after consuming the HCHF diet, common carp presented elevated serum T‐CHO levels. Sufficient BA levels are necessary for the intestinal digestion of lipids. To fulfill this requirement, the hepatopancreas enhances the conversion of cholesterol into BAs, which depletes hepatopancreatic T‐CHO, increases TBA levels, and lowers LDL‐C to reduce cholesterol from the hepatopancreas to systemic tissues. To counter the decrease in cholesterol within the hepatopancreas, the fish raised their serum HDL‐C levels, aiding cholesterol transport back to the liver. Moreover, the expression of the endogenous cholesterol‐producing gene *hmgcr* in the fish decreased to manage diet‐induced dyslipidemia and decrease T‐CHO and LDL‐C accumulation in the serum. The absence of significant changes in hepatopancreatic T‐CHO levels, despite altered *hmgcr* expression and BA profiles, may reflect the dynamic balance between cholesterol synthesis, consumption for BA production, and uptake from the circulation. This homeostasis appears to be maintained even under the metabolic stress of the HCHF diet. However, this phenomenon was attenuated following probiotic supplementation. Similar results have been reported in mammals. LP FZU3013 can increase BA circulation, attenuate body weight gain induced by a high‐fat diet, and decrease the serum levels of TG, T‐CHO, and LDL‐C [53]. LP LP104 can reduce the serum TC, TG, and LDL‐C levels in mice fed a high‐fat diet; activate the hepatic expression of AMPK and proteins associated with fatty acid oxidation and transport; and thereby ameliorate glycolipid metabolic disorders [54].

FXR is essential for controlling the metabolic process of glycolipid metabolic diseases, which can result from the HCHF diet. Research conducted at both the individual and cellular levels has demonstrated that Glu enhances the FXR expression. For example, D‐Glu has been shown to upregulate the FXR gene expression in primary rat hepatocytes [55]. In *Megalobrama amblycephala*, high‐carbohydrate diets resulted in elevated hindgut FXRα transcript levels [56]. Similarly, the present study demonstrated that common carp fed the HCHF diet presented increased hepatopancreatic FXR expression. FXR generally suppresses hepatic gluconeogenesis by upregulating the expression of SHP while downregulating the expression of HNF4α, FOXo1, and their target genes, including *pck1*, *fbp*, and *g6pase* [23]. In the present study, SHP inhibited *hnf4α* and its downstream gene *pck1*, which aligns with findings reported in mammals [23]. However, there was an increase in the expression of the *foxo1* gene, which lies downstream of the SHP. This observation may be attributed to the limited capacity of fish to regulate FOXo1, which serves as a negative regulator of the insulin PI3K/AKT signaling pathway, thereby diminishing insulin effectiveness in regulating carbohydrate metabolism due to inadequate insulin synthesis [6, 57]. Under pathological circumstances, such as obesity and T2DM, the transcriptional activity of FOXo1 is increased, which can facilitate hepatic gluconeogenesis and TG production [58]. In this study, the HCHF diet resulted in impaired hepatocyte function in common carp. Coupled with the low carbohydrate tolerance and insufficient insulin secretion in fish, this led to a diminished expression level of the insulinogenin gene and limited insulin synthesis. Consequently, there was a significant increase in *foxo1* gene expression, which was associated with increased PEPCK enzyme activity, correlating with sustained high‐Glu levels for an extended duration. Probiotic supplementation in the present study significantly decreased the activities of the hepatopancreatic gluconeogenic enzymes PEPCK and G6Pase as well as the expression of genes associated with gluconeogenesis and Glu transport. This consequently inhibited hepatic Glu uptake and transport and improved Glu metabolic capacity in common carp. Consistent with this, the elevated hepatopancreatic ALT activity under HCHF feeding may reflect increased transamination and gluconeogenic flux in hepatocytes under metabolic stress, and its reduction in the probiotic‐supplemented groups is consistent with alleviation of this metabolic burden, supporting a partial improvement in hepatopancreatic metabolic status at the tissue level.

Excess glycolipid intake is converted into fatty acids in the liver, which are subsequently stored as TGs. The primary substrate for fatty acid synthesis is acetyl‐CoA. First, saturated fatty acids are generated via the enzymatic activity of ACC and FAS. Unsaturated fatty acids are subsequently synthesized under the action of saturated fatty acid dehydrogenase. In the final stages, unsaturated fatty acids are activated to form fatty acids, leading to the synthesis of TGs. FXR contributes to the downregulation of fatty acid synthesis. The activation of hepatic FXR/SHP serves to inhibit SREBP‐1c expression and decelerates the transcription of genes related to fatty acid synthesis, such as ACC1 and FAS [59]. This study examined the effects of HCHF on the hepatopancreas, muscle, and whole‐body nutritional composition of common carp. Moreover, dietary interventions resulted in elevated TG and crude fat contents, accompanied by a decrease in the expression of lipid‐synthesizing genes, including *srebp1*, *acc1*, and *fas*. This phenomenon may be attributed to the excessive consumption of glycolipids, which leads to the deposition of fat within the fish. The fish reduced endogenous fatty acid synthesis through negative feedback mechanisms. The findings presented here, which focused on Nile tilapia [60] and grass carp [61], corroborate the results of this study. High‐fat diets adversely affect the functionality of fish hepatocytes, resulting in a compromised fatty acid β‐oxidation system and reduced lipolysis. Furthermore, elevated concentrations of polyunsaturated fatty acids within high‐fat diets can further diminish β‐oxidation activity [62]. Moreover, FXR regulates PPARs and their cotranscriptional activator, PGC‐1α, in fatty acid β‐oxidation. CD36 is a principal protein associated with fatty acid transport. In a MAFLD mouse model, the overexpression of CD36 was linked to increased fatty acid uptake by the liver [63]. Similarly, increased serum CD36 levels in obese individuals can precipitate hepatic steatosis [64]. Our work revealed that the HCHF diet decreased the expression of genes linked to β‐oxidation and lipolysis while concurrently increasing the expression of *cd36* genes in the hepatopancreas, except for *cpt1*. In conjunction with the results derived from Oil Red O and HE staining, these outcomes may be associated with the HCHF diet inducing abnormal hepatocyte function and diminishing β‐oxidation activity. This chain of events is associated with disturbances in glycolipid metabolism in common carp, impacting metabolism‐related regulatory pathways and correlating with fat deposition. Probiotics exhibit the same regulatory pathways for lipid metabolism in fish as they do in mammals. Studies have demonstrated that in mice, *Pediococcus pentosaceus* PP04 promotes lipid catabolism by upregulating PPARα; inhibits fatty acid synthesis by downregulating SREBP‐1c, FAS, and stearoyl‐CoA desaturase‐1 (SCD1); and ameliorates high‐fat diet‐induced hyperlipidemia via the AMPK signaling pathway [65]. Similarly, in the present study, probiotic supplementation decreased serum lipid levels and the expression of fatty acid synthesis‐related genes and reduced hepatopancreatic lipid deposition.

A stable gut microbial community is essential for the host’s health. The gut microbiota is influenced by dietary practices, environmental variables, and genetic predispositions. The composition of the gut microbiota can be considerably altered by high‐carbohydrate or high‐fat diets. In blunt snout bream, studies have indicated that such diets increase the relative abundance of *Fusobacteria* and *Proteobacteria* while decreasing that of *Firmicutes* and *Bacteroidetes* [66]. High‐fat diet‐fed largemouth bass presented a decrease in gut microbiota diversity, characterized by an increase in the prevalence of *Firmicutes* and potentially harmful bacteria, along with a decrease in beneficial bacteria such as *Fusobacteria* [67]. According to our research, the HCHF diet decreased the diversity of the gut microbiota and changed the structural composition of common carp. Specifically, the abundance of gram‐negative pathogenic bacteria, including *Serratia*, *Pseudomonas*, *Psychrobacter*, and *Pantoea*, was elevated in HCHF diet‐fed fish. Numerous studies have reported that probiotics inhibit the growth and proliferation of pathogenic bacteria by competing with pathogenic microorganisms for binding sites and nutrients on intestinal epithelial cells. Alternatively, they strengthen the intestinal barrier and maintain gut microbiota homeostasis by producing antimicrobial substances (e.g., bacteriocins, organic acids, polyphenols, and polypeptides) and enhancing the tight junctions between intestinal epithelial cells. Although full‐length *16S* rRNA sequencing provides improved taxonomic resolution compared with short‐read approaches, species‐level identification remains tentative. With this consideration, the abundance of *C. somerae*, an indigenous intestinal probiotic in fish, significantly increased in both the LP and BL groups. The probiotic effects of *C. somerae* in fish have been extensively documented. The fermentation products of *C. somerae* XMX‐1 improve intestinal and hepatic health and reduce hepatic lipid deposition in carp [68]. Dietary supplementation with *C. somerae* XMX‐1 ameliorates hepatic and intestinal health and enhances antiviral immunity in zebrafish [69]. *C. somerae* can metabolize vitamin B12 to strengthen interspecies interactions among intestinal microbes and the integrity of intestinal barrier tight junctions, thereby increasing host resistance to pathogenic infection [70]. *C. somerae* CPU‐CS01, which is isolated from the gut of healthy crucian carp, confers increased resistance to hydrogen peroxide‐induced oxidative stress and *Aeromonas hydrophila* infection [71]. Additionally, *C. somerae* can activate the parasympathetic nervous system via its metabolite acetic acid, thereby promoting insulin secretion and Glu utilization capacity in zebrafish [72]. In the present study, probiotic supplementation ameliorated glycolipid metabolic disorders, which may be associated with the increased abundance of *C. somerae* and other intestinal probiotics, as well as the comprehensive probiotic effects exerted by these microbes. Direct colonization of the supplemented strains was not verified in this study. Nevertheless, probiotics can modulate host metabolism through transient interactions with the resident microbiota without requiring permanent engraftment. The observed enrichment of *C. somerae* and potentially BSH‐active taxa supports this functional‐shift mechanism. Future studies using strain‐specific qPCR are needed to confirm colonization and persistence.

Probiotic supplementation was associated with altered BA metabolism and enterohepatic dynamics, which can modulate the systemic metabolism and health. *Bifidobacterium breve* has been shown to increase the abundances of *Bifidobacterium* and *Clostridium sensu stricto* 1, promote the production of unconjugated BAs in the gut of colitis mice, and increase CA levels. CA can inhibit tumor necrosis factor‐α (TNF‐α)‐mediated inflammatory responses, upregulate the expression of occludin, claudin‐1, and MUC2; and thereby improve gut health [73]. LP ATCC8014 ameliorates abnormal BA metabolism and hypercholesterolemia by remodeling the gut microbiota [74]. In the present study, probiotic supplementation significantly increased the contents of unconjugated BAs with FXR‐activating effects (CDCA, DCA, and LCA) in the hepatopancreas. These observations are consistent with a model in which probiotic supplementation modulates BA metabolic gene expression, coinciding with improvements in glycolipid metabolic parameters. Although both LP and BL alleviated HCHF‐induced glycolipid disorders, and several differences in their regulatory patterns were observed. At the gut microbiota level, LP‐enriched *Clostridium*, whereas BL enriched *Bacillus*, both genera known for potential BSH activity, suggesting distinct ecological niches. At the BA level, BL exerted stronger modulation, affecting more BA species than LP, likely due to its spore‐forming nature and superior enzyme‐producing capacity. Consistently, the activation of hepatic FXR/SHP appeared to be more pronounced in the BL group. These observations suggest that while the gut microbiota–BA–FXR axis represents a common pathway, the specific effects are strain‐dependent, with spore‐forming Bacillus potentially offering advantages in metabolic regulation. However, direct comparisons are complicated by inherent physiological differences, and the precise molecular determinants warrant further investigation. Future studies employing FXR antagonists or gene knockout models would be valuable to dissect the causal relationship between the gut microbiota–BA–FXR axis and the differential metabolic outcomes induced by these two probiotics.

Gut microbiota metabolites play critical regulatory roles in glycolipid metabolism. A meta‐analysis involving biomarker validation in 345 patients with T2DM revealed that these biomarkers were associated with gut microbiota dysbiosis and reduced abundances of intestinal butyrate‐producing bacteria [75]. High‐Glu dietary intake enhances Glu utilization by the gut microbiota, which undergoes rapid fermentation via the glycolytic pathway to generate energy, accompanied by the accumulation of organic acids. This metabolic activity may inhibit certain beneficial bacteria while promoting the proliferation of saccharophilic bacteria (e.g., *Escherichia coli*). When excessive protein intake is coupled with a HCHF diet, intestinal pathogenic bacteria may excessively catabolize amino acids to produce amines and phenols; these substances exhibit cytotoxicity and can induce intestinal inflammation. Taken together, these findings indicate that intestinal metabolites are closely associated with the host’s health. The results of the present study revealed that following probiotic supplementation, numerous metabolism‐related signaling pathways were significantly enriched in the gut of common carp, indicating that probiotics increase the intestinal capacity for nutrient digestion, absorption, and metabolism by remodeling the intestinal ecology and reestablishing intrinsic intestinal metabolic homeostasis. Increased abundances of beneficial intestinal bacteria and their beneficial metabolites positively regulate host health.

## 5. Conclusion

This study employed common carp fed a HCHF diet as a model and investigated the regulatory effects and underlying mechanisms of dietary LP and BL on host glycolipid metabolic disorders. Our results indicate that both probiotics alleviated HCHF‐induced glycolipid disorders, partly through modulation of BA metabolism and lipid metabolism‐related gene expression, leading to reduced hepatopancreatic lipid deposition. Furthermore, probiotics significantly increased the abundance of the indigenous beneficial intestinal bacterium *C. somerae* and altered the host’s intestinal metabolic profile. These findings offer a theoretical reference for understanding the gut microbiota–BA axis regulatory network in aquatic animals and provide practical insights for probiotic application in HCHF feed formulations for intensive common carp farming (Figure 15).

**Figure 15:**
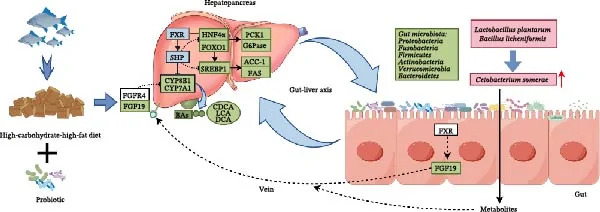
Graphical abstract. *L. plantarum* and *B. licheniformis* alleviate glycolipid metabolism disorders in common carp in association with the gut microbiota–bile acid metabolism axis.

**Nomenclature**
BAs: Bile acids
BSH: Bile salt hydrolase
CAT: Catalase
CYP7A1: Cholesterol 7α‐hydroxylase
CYP8B1: Sterol 12α‐hydroxylase
FGF15/19: Fibroblast growth factor 15/19
FXR: Farnesoid X receptor
G6Pase: Glucose‐6‐phosphatase
Glu: Glucose
HCHF: High‐carbohydrate–high‐fat
HDL‐C: High‐density lipoprotein cholesterol
HK: Hexokinase
LDL‐C: Low‐density lipoprotein cholesterol
MAFLD: Metabolic dysfunction‐associated fatty liver disease
MDA: Malondialdehyde
PEPCK: Phosphoenolpyruvate carboxykinase
PK: Pyruvate kinase
SHP: Small heterodimer partner
T2DM: Type 2 diabetes mellitus
TBA: Total bile acid
T‐CHO: Total cholesterol
TG: Triglyceride
T‐SOD: Total superoxide dismutase.

## Author Contributions

**Peng Pang**: investigation, writing – original draft, funding acquisition. **Xiao Yan**: conceptualization, writing – review and editing, methodology, funding acquisition. **Xin Long**: data curation, formal analysis. **Jiali Mi**: visualization. **Chaobin Qin**: supervision. **Liping Yang**: methodology. **Guoxing Nie**: project administration, supervision, funding acquisition.

## Funding

This study was supported by the Natural Science Foundation of Henan (Grant 242300421114), the National Natural Science Foundation of China (Grant 32373142), the Scientific Research and Practical Innovation Project of Henan Normal University (Grant YB202503), and the Special Fund for Henan Agriculture Research System (Grant HARS‐22‐16‐S).

## Ethics Statement

This article does not contain any experiments conducted by the authors with human participants. All experiments were conducted following standard procedures.

## Conflicts of Interest

The authors declare no conflicts of interest.

## Supporting Information

Additional supporting information can be found online in the Supporting Information section.

## Supporting information


**Supporting Information** Table S1: The nutritional quantity and composition of the experimental diets. Table S2: Enzyme activities and biochemical assay information. Table S3: The primers of quantitative real‐time PCR. Table S4: Antibodies used for Western blotting. Table S5: Effect of the HCHF diet on growth performance of common carp. Table S6: Effects of probiotics on growth performance of common carp. Table S7: Significantly altered bile acids in the LP and BL groups. Table S8: Significantly upregulated differential metabolites in positive and negative ion modes in the LP group. Table S9: Significantly upregulated differential metabolites in positive and negative ion modes in the BL group.

## Data Availability

The *16S* rRNA sequencing data of the gut microbiota in this study are available in the NCBI SRA database. The accession link is https://www.ncbi.nlm.nih.gov/bioproject/PRJNA1467536, with the Reference Number PRJNA1467536.
