# Supplementary material for: Lactobacillus plantarum and Bacillus licheniformis Ameliorate High‐Carbohydrate–High‐Fat‐Induced Glycolipid Disorders in Common Carp in Association With the Gut Microbiota and Bile Acid Axis
Source: Aquac Nutr. 2026 Sep 29;2026:6145147. doi: 10.1155/anu/6145147 (PMC13624548; doi:10.1155/anu/6145147)
Supplement: Supplementary file 1 [file ANU-2026-6145147-s001.docx]

**Supplementary information**

***Lactobacillus plantarum and Bacillus licheniformis ameliorate high-carbohydrate-high-fat-induced glycolipid disorders in common carp in association with the gut microbiota and bile acid axis***

Peng Pang, Xiao Yan^*^, Xin Long, Jiali Mi, Chaobin Qin, Liping Yang, Guoxing Nie^*^

*College of Fisheries, Henan Normal University, No. 46 Jianshe Road, Xinxiang 453007, Henan Province, P.R. China*

**^*^*These authors contributed equally to this work and share corresponding authorship:***

**Guoxing Nie**, E-mail: [*niegx@htu.cn*](mailto:niegx@htu.cn), ORCID: <https://orcid.org/0000-0002-0758-9933>

**Xiao Yan**, E-mail: [*yanxiaobangong@163.com*](mailto:yanxiaobangong@163.com), ORCID: <https://orcid.org/0000-0001-7626-2128>

**Table S1 The nutritional quantity and composition of experimental diets**

| Component (%) | Groups | |
| --- | --- | --- |
|  | CON | HCHF |
| Fish meal | 30.00 | 30.00 |
| Corn protein powder | 19.00 | 19.00 |
| Wheat meal | 11.80 | 11.00 |
| Corn Starch | 15.00 | 29.00 |
| Fish oil | 2.00 | 7.60 |
| Monocalcium phosphate | 1.00 | 1.00 |
| Vitamin Premix ^a^ | 0.07 | 0.07 |
| Mineral Premix ^b^ | 0.10 | 0.10 |
| Choline chloride | 0.20 | 0.20 |
| Carboxy methyl cellulose | 1.00 | 1.00 |
| Microcrystalline Cellulose | 19.83 | 1.03 |
| Proximate composition, measured value (%, wet matter) | | |
| Dry matter | 92.86 | 92.55 |
| Crude protein | 35.60 | 33.87 |
| Crude lipid | 6.43 | 12.78 |
| Ash | 6.85 | 6.70 |

^a^Vitamin premix (per kg diet): VA 2,000,000-6,000,000 IU, VB₁ 550-1,800 mg, VB₂ 860-2,880 mg, VB₆ 5,500-18,000 mg, VB₁₂ 10-20 mg, VD₃ 1,000,000-2,000,000 IU, VE 9,000-30,000 mg, VK₃ 2,700-9,000 mg, nicotinamide 10,000-42,000 mg, folic acid 900-3,000 mg, calcium pantothenate 5,500-18,000 mg, biotin 20-60 mg, VC 6,000-20,000 mg, inositol 7,200-24,000 mg.

^b^Mineral premix (per kg diet): Cu 3,000-12,000 mg, Fe 5,000-20,000 mg, Zn 8,000-32,000 mg, Mn 10,000-40,000 mg, I 200-800 mg, Se 100-400 mg.

***Growth performance***

The growth and feed utilization parameters were calculated as follows:

Final body weight (FBW, g) = total final body weight/final number of fish.

Weight gain rate (WG, %) = [final body weight (g) – initial body weight (g)]/initial body weight (g) × 100.

Survival rate (SR, %) = (final number of fish/initial number of fish) × 100.

Hepatosomatic index (HSI, %) = [hepatopancreas weight (g)/body weight (g)] × 100.

Visceral somatic index (VSI, %) = [visceral weight (g)/body weight (g)] × 100.

Feed conversion ratio (FCR) = feed intake (g)/[final body weight (g) – initial body weight (g)].

Specific growth rate (SGR, %/day) = [ln(final body weight, g) – ln(initial body weight, g)] × 100/number of feeding days.

Condition factor (CF, %) = [final body weight (g)/length³ (cm)] × 100.

**Table S2 Enzyme activities and biochemical assays information**

| **Abbreviation** | **Full name** | **Catalog No.** | **Manufacturer** |
| --- | --- | --- | --- |
| GLU | Glucose | 100000240 | BIOSINO, China |
| TG | Triglyceride | A110-2-1 | NJJCBIO, China |
| T-CHO | Total cholesterol | A111-2-1 | NJJCBIO, China |
| HDL-C | High-density lipoprotein cholesterol | A112-2-1 | NJJCBIO, China |
| LDL-C | Low-density lipoprotein cholesterol | A113-2-1 | NJJCBIO, China |
| TBA | Total bile acid | E003-1-1 | NJJCBIO, China |
| MDA | Malondialdehyde | A003-1-2 | NJJCBIO, China |
| ALT | Alanine aminotransferase | C009-1-1 | NJJCBIO, China |
| AST | Aspartate aminotransferase | C010-1-1 | NJJCBIO, China |
| T-SOD | Total superoxide dismutase | A001-1-2 | NJJCBIO, China |
| CAT | Catalase | A007-1-1 | NJJCBIO, China |
| LD | Lactate | A019-2-1 | NJJCBIO, China |
| HK | Hexokinase | BC0740 | Solarbio, China |
| PK | Pyruvate kinase | BC0540 | Solarbio, China |
| PEPCK | Phosphoenolpyruvate carboxykinase | BC3310 | Solarbio, China |
| G6Pase | Glucose-6-phosphatase | BC3320 | Solarbio, China |

**Table S3 The primers of quantitative real-time PCR**

| **Gene** | **Sequences (5′-3′) (sense)** | **Sequences (5′-3′) (antisense)** | **GenBank ID** |
| --- | --- | --- | --- |
| *18S (18S ribosomal RNA)* | F: GAGACTCCGGCTTGCTAAAT | R: CAGACCTGTTATTGCTCCATCT | LN591197.1 |
| *fxr (farnesoid X receptor)* | F: CCCCTACAGCCATCAGT | R: GACCGACAATGCTCCCT | XM_042729372.1 |
| *rxra (retinoid X receptor alpha)* | F: CACCCAATGATCCAGTCACAAACA | R: AGCTCATTCCATCCTGCTCGTAGA | XM_019094166.2 |
| *cyp7a1 (cholesterol 7 alpha-hydroxylase)* | F: CTACCTGACCCTCTTTGG | R: CGTGGAGCATTGTCTTGG | XM_042718130.1 |
| *cyp8b1 (sterol 12 alpha-hydroxylase)* | F: CTTGCCATTGCTTGTCAT | R: GTATTCGGCTATCGTTCC | XM_019073965.2 |
| *shp (small heterodimer partner)* | F: GAAGTTCTGGAGTCTGGATT | R: GGATGAAGAGGCACAAGG | XM_042741839.1 |
| *hnf4α (hepatocyte nuclear factor-4α)* | F: CAGGATTAGCACCAGAAGAT | R: TGTCGCCATTCAAGATAGG | XM_019071428.2 |
| *foxo1 (forkhead box protein O1)* | F: ACGGCTCACCTTATCTCA | R: CTTGCTGTTGTTGTCCATAG | XM_042739020.1 |
| *fgf19 (fibroblast growth factor 19)* | F: GTGGGAAAACTGTATGGATCGC | R: AAGGGCTCCATGTTTGCTTG | XM_042728520.1 |
| *fgfr4 (fibroblast growth factor receptor 4)* | F: CTACGCAACTTCACTATCACA | R: CCAGCATCATCAAGACCATT | XM_042747649.1 |
| *hmgcr (3-hydroxy-3-methylglutaryl coenzyme A reductase)* | F: TGTCTGTCCTGGCGAACTAC | R: GCTTGTTGTCCTCTTCCTCCT | XM_042724647.1 |
| *tgr5 (transmembrane G protein-coupled receptor 5)* | F: GCATTACGAACGCTACCTGTG | R: TGGAAGAGTTACGAGATGTGGT | XM_042764258.1 |
| *sglt1 (recombinant sodium/glucose cotransporter 1)* | F: CTAAAGAAGAGGAGGCAGAGTTG | R: ACAGACGGTGAGGAGGATAATA | JN867793.1 |
| *glut2 (glucose transporter 2)* | F: GAGGGTCTTTGTGGGAACTATG | R: GTTTCAGGTACACGCAAGTAGA | XM_042718276.1 |
| *cck (cholecystokinin)* | F: TCTCCACCAAAGGCACATATC | R: ATCCATCCATCCCAAGTAATCTC | XM_019117897.2 |
| *hk (hexokinase)* | F: CGTTGTAGCCGTAGTGAATGA | R: GCTACCCGTTCCTGCAATTA | XM_019097065.2 |
| *pfk (phosphofructokinase)* | F: CGTTCGACAGGAATTTTGGC | R: TTCATGCCGATCACACAAGC | XM_042766957.1 |
| *pk (pyruvate kinase)* | F: GATATGGGCTCTGCCTTCAT | R: TCCGATGGTGCAGATGATTC | XM_042744551.1 |
| *g6pase (glucose-6-phosphatase)* | F: GAGGCCTTCAACAGACAGAAA | R: GAGCTTTGAGAAGCAGGTACAA | AF427863.1 |
| *pck1 (phosphoenolpyruvate carboxykinase 1)* | F: CGTTGGAGAGCGGAGAGTTC | R: ATGTGAGCGATGAGCGTGAG | KP250869.1 |
| *fbp (fructose 1,6-bis phosphatase)* | F: CCTGCCATCGGTGAGTTTAT | R: CCATCCTCTGGGAATTTCTTCT | AF427864.1 |
| *gys (glycogen synthase)* | F: TTTACTACACATGCCACCCTAC | R: TCTCTCCTGCCTCTTGATCTAT | XM_019090903.1 |
| *pygl (phosphorylase glycogen)* | F: TGGTTGACGACGATGCTTTC | R: ACTGCGCAAACTTCAGCTTG | XM_042737361.1 |
| *pi3k (phosphoinositide3-kinase)* | F: GAAGATGACGACTGGAGAG | R: GCCTGTAGTGACTGATGAG | XM_019085737.2 |
| *akt (protein Kinase B)* | F: ACTGTTATTGAGCGCACCTT | R: TCCATTGGCTCCTCCTCTTC | XM_042774924.1 |
| *gsk3β (glycogen synthase kinase 3β)* | F: ATCAGGTGCATGAGGAGTTGAG | R: AGCAGAGCATTTCCAAACGC | XM_042774357.1 |
| *gcga (glucagon a)* | F: TTCTGAGGGAACTTTCTCCAAC | R: GTTCATGAGCCACTGAACAAAG | XM_019065527.2 |
| *ins (insulin)* | F: GTGGTCCAACAGGTTTCTTCTA | R: AAGTCAGCCACCTCAGTTTC | XM_019099888.2 |
| *glp1α (glucagon-like peptide 1α)* | F: TCACGCTGAGGGAACCTA | R: GCTTGGGCTGTCCAGATT | XM_019094634.2 |
| *igf1 (insulin-like growth factor 1)* | F: GGATATGGGCCTAGTTCAAGAC | R: TACGGGTGCACAATACATCTC | XM_042740819.1 |
| *acc1 (acetyl CoA carboxylase 1)* | F: GTCACTGGCGTATGAGGATATT | R: TCCACCTGTATGGTTCTTTGG | XM_042757417.1 |
| *fas (fatty acid synthase)* | F: GACAGGCCGCTATTGCTATT | R: TGCCGTAAGCTGAGGAAATC | GQ466045.1 |
| *srebp1 (sterol regulatory element-binding protein 1)* | F: CGTCTGCTTCACTTCACTACTC | R: GGACCAGTCTTCATCCACAAA | XM_042730308.1 |
| *pparα (peroxisome proliferator-activated receptor α)* | F: GGGAAAGAGCAGCACGAG | R: GCGTGCTTTGGCTTTGTT | FJ849065.1 |

**Continued Table S3:**

| Gene | Sequence (5′-3′) (sense) | Sequences (5′-3′) (antisense) | GenBank ID |
| --- | --- | --- | --- |
| *pparγ (peroxisome proliferator-activated receptor γ)* | F: AAGTCACCGAATTCGCCAAG | R: TGCCGTCTTTGTTCATGAGG | FJ849064.1 |
| *hsl (hormone-sensitive lipase)* | F: GCAGGTGGTAACCTGTGTATAA | R: TTAGCAGAGTTGCTGGGTATG | XM_042776500.1 |
| *lpl (lipoprotein lipase)* | F: CGCTCCATTCACCTGTTCAT | R: GCTGAGACACATGCCCTTATT | FJ716101.1 |
| *cpt1 (carnitine palmitoyltransferase 1)* | F: CAGATGGAAAGTGTTGCTAATGAC | R: TGTGTAGAAGTTGCTGTTGACCA | JQ361077.1 |
| *fatp1 (fatty acid transport protein 1)* | F: AATCAGCCGAAGAAGGACAC | R: AATCCACAGGCACCCGTCTT | XM_019081506.1 |
| *cd36 (cluster of differentiation 36)* | F: CACTTCCTCTATGGCAGCAATG | R: CCGTCTCATCGTCCAGAACT | XM_019078777.2 |
| *creb (cAMP response element binding protein)* | F: GAGACAGACACTCAGCAGATTG | R: TGGATGACGCCGTGAACTT | XM_019107844.2 |
| *pgc1α (peroxisome proliferator-activated receptor γ coactivator 1α)* | F: TGCTGCTTTGGTTGGTGAAG | R: TGCATCGAGGTCACTGACATC | AB767302.1 |
| *ampkα1* *(adenosine mono‐phosphate activated protein kinase α1)* | F: TGCGAGAAGTTTGAGTGCAC | R: TTCATAATGCGCCGGTTGTC | XM_019104472.2 |
| *ampkα2 (adenosine mono‐phosphate activated protein kinase α2)* | F: AAATGTGTGCCAGCCTCATC | R: ATGTTTGTGGCGCTACTGTG | XM_042777568.1 |
| *asbt (apical sodium-dependent bile acid transporter)* | F: TCCTCAACGTGGTGATGAGC | R: AGCACACCACCTATGACTGC | XM_019119219.2 |
| *ostα (organic solute transporter α)* | F: TCTTGTTTGGGCATGTGGGT | R: TTGCAGCTGGCTGAGAATCA | XM_042714434.1 |

**Table S4 Antibodies used for Western blotting**

| **Target protein** | **Catalog No.** | **Host** | **Dilution** | **Manufacturer** |
| --- | --- | --- | --- | --- |
| FXR (NR1H4) | bs-12867R | Rabbit | 1:1000 | BIOSS, China |
| SHP (NR0B2) | bs-4311R | Rabbit | 1:1000 | BIOSS, China |
| phospho-AMPKα (Thr172) | 2535 | Rabbit | 1:1000 | CST, USA |
| AMPKα | 5831 | Rabbit | 1:1000 | CST, USA |
| β-actin | 81115-1-RR | Rabbit | 1:20000 | Proteintech, China |

**Table S5 Effect of the HCHF diet on growth performance of common carp**

| Index | Groups | |
| --- | --- | --- |
|  | CON | HCHF |
| IBW (g) | 7.44 ± 1.31 | 7.42 ± 1.49 |
| FBW(g) | 83.78 ± 1.44 | 91.48 ± 0.23^*^ |
| WG (%) | 1026.19 ± 21.40 | 1133.18 ± 3.40^*^ |
| SGR (%/d) | 3.46 ± 0.03 | 3.59 ± 0.00^*^ |
| FCR | 1.22 ± 0.02 | 1.11 ± 0.00^*^ |
| HSI (%) | 3.99 ± 0.16 | 4.88 ± 0.35^*^ |
| VSI (%) | 13.44 ± 1.69 | 13.35 ± 1.77 |
| CF (g/cm^3^) | 2.59 ± 0.07 | 2.50 ± 0.06 |

Note: All information was represented as mean values ± S.E.M. Significant differences are indicated by “*” (* *P* < 0.05; ** *P* < 0.01; *** *P* < 0.001).

**Table S6 Effects of probiotics on growth performance of common carp**

| Index | Groups | | | |
| --- | --- | --- | --- | --- |
|  | HCHF | LP | BL |  |
| IBW (g) | 47.90 ± 0.30 | 47.91 ± 0.28 | 47.85 ± 0.27 |  |
| FBW (g) | 203.36 ± 1.94 | 203.70 ± 0.43 | 197.19 ± 1.75 |  |
| WG (%) | 324.51 ± 4.38 | 325.21 ± 0.59 | 312.09 ± 3.32 |  |
| SGR (%/d) | 2.30 ± 0.02 | 2.30 ± 0.00 | 2.25 ± 0.01 |  |
| CF (g/cm^3^) | 2.61 ± 0.07 | 2.62 ± 0.07 | 2.71 ± 0.06 |  |
| FCR | 1.14 ± 0.02 | 1.14 ± 0.00 | 1.19 ± 0.01 |  |
| HSI (%) | 2.26 ± 0.13^a^ | 2.36 ± 0.20^a^ | 1.70 ± 0.13^b^ |  |
| VSI (%) | 9.39 ± 0.31 | 9.45 ± 0.29 | 9.44 ± 0.28 |  |

Note: Mean values ± S.E.M. Different lowercase letters of paired data superscript indicate significantly different (P < 0.05).

**Table S7 Significantly altered bile acids in the LP and BL groups**

| **Index** | **Compounds** | **Class** | **Classified by source** | **Classified by structure** | **VIP** | **Pvalue** | **FDR** |
| --- | --- | --- | --- | --- | --- | --- | --- |
| 23-DCA | Nor-Deoxycholic Acid | BAs | Secondary bile acid | Free bile acid | 0.2915 | 1.0730E-13 | 1.7558E-13 |
| MDCA | murideoxycholic acid | BAs | Secondary bile acid | Free bile acid | 0.9140 | 1.5021E-21 | 6.0084E-21 |
| 3-oxo-DCA | 3-oxodeoxycholic acid | BAs | Secondary bile acid | Free bile acid | 0.9042 | 2.1731E-25 | 1.5647E-24 |
| 12-KLCA | 12-ketolithocholic acid | BAs | Secondary bile acid | Free bile acid | 0.9797 | 1.1369E-20 | 2.9234E-20 |
| 3β-CA | 3β-Cholic Acid | BAs | Primary bile acid | Free bile acid | 0.9303 | 4.4559E-29 | 8.0206E-28 |
| α-MCA | α-muricholic acid | BAs | Secondary bile acid | Free bile acid | 0.9384 | 2.0597E-28 | 2.0554E-27 |
| GUDCA-3S | Glycoursodeoxycholic Acid 3 Sulfate Sodium | BAs | Secondary bile acid | Free bile acid | 0.1643 | 3.8499E-21 | 1.2600E-20 |
| 7-KDCA | 7-Ketodeoxycholic acid | BAs | Primary bile acid | Free bile acid | 1.2946 | 9.1155E-21 | 2.5243E-20 |
| CA | cholic acid | BAs | Primary bile acid | Free bile acid | 0.9687 | 7.6373E-21 | 2.2912E-20 |
| CDCA | Chenodeoxycholic acid | BAs | Primary bile acid | Free bile acid | 1.5622 | 4.5543E-23 | 2.3422E-22 |
| CDCA-3S | chenodeoxycholic acid3-sulfate disodium salt | BAs | Secondary bile acid | Free bile acid | 1.1616 | 3.5406E-23 | 2.1243E-22 |
| LCA | Lithocholic acid | BAs | Secondary bile acid | Free bile acid | 1.5489 | 9.2526E-09 | 1.0409E-08 |
| 3-oxo-CA | 3-Oxocholic acid | BAs | Secondary bile acid | Free bile acid | 1.3391 | 1.1438E-12 | 1.6470E-12 |
| DHCA | Dehydrocholic acid | BAs | Secondary bile acid | Free bile acid | 1.5655 | 2.4416E-19 | 5.4935E-19 |
| 7-KLCA | 7-ketolithocholic acid | BAs | Secondary bile acid | Free bile acid | 1.2308 | 2.2536E-19 | 5.4087E-19 |
| 12-oxo-CDCA | 12-Oxochenodeoxycholic acid | BAs | Primary bile acid | Free bile acid | 1.1954 | 2.2837E-28 | 2.0554E-27 |
| CA-3S | Cholic Acid 3 Sulfate Sodium Salt | BAs | Secondary bile acid | Free bile acid | 1.2892 | 1.4581E-18 | 3.0877E-18 |
| DCA | Deoxycholic acid | BAs | Primary bile acid | Free bile acid | 1.1253 | 1.2717E-10 | 1.5787E-10 |
| CDCA-3Gln | Chenodeoxycholic acid-3-β-D-glucuronide | Glucuronide | Primary bile acid | Conjugated bile acid | 1.2965 | 9.2267E-22 | 4.1520E-21 |
| GCDCA | Glycochenodeoxycholic acid | Glyco | Secondary bile acid | Conjugated bile acid | 0.1116 | 6.3853E-09 | 7.4152E-09 |
| Tω-MCA | Tauro-ω-muricholic Acid sodium salt | Tauro | Primary bile acid | Conjugated bile acid | 0.7213 | 3.5752E-13 | 5.3629E-13 |
| TCA-3S | Taurocholic Acid 3 sulfate sodium salt | Tauro | Primary bile acid | Conjugated bile acid | 1.5173 | 7.8594E-12 | 1.0479E-11 |
| TCDCA | Taurochenodeoxycholic acid | Tauro | Primary bile acid | Conjugated bile acid | 0.0810 | 1.8231E-14 | 3.2815E-14 |
| THCA | Taurohyocholic acid | Tauro | Secondary bile acid | Conjugated bile acid | 1.1836 | 7.9176E-16 | 1.5835E-15 |
| TLCA | taurolithocholic acid | Tauro | Secondary bile acid | Conjugated bile acid | 0.4322 | 1.1948E-13 | 1.8702E-13 |
| TLCA-3S | taurolithocholic acid-3-sulfate | Tauro | Secondary bile acid | Conjugated bile acid | 1.0825 | 1.5224E-08 | 1.6608E-08 |
| TUDCA | Tauroursodeoxycholic acid | Tauro | Secondary bile acid | Conjugated bile acid | 0.0228 | 2.9194E-15 | 5.5314E-15 |
| THDCA | Taurohyodeoxycholic Acid (sodium salt) | Tauro | Secondary bile acid | Conjugated bile acid | 0.5398 | 1.0533E-10 | 1.3543E-10 |

**Table S8 Significantly up‑regulated differential metabolites in positive and negative ion modes in LP group**

| **ID** | **foldChange** | **log2FoldChange** | **pvalue** | **FDR** | **vip** | **m/z** | **Name** | **type** |
| --- | --- | --- | --- | --- | --- | --- | --- | --- |
| M307 | 1.510369527 | 0.594901563 | 0.000755085 | 0.008664375 | 1.481540328 | 282.2799 | Sphingosine | POS |
| M2304 | 1.26997364 | 0.344798552 | 0.001217559 | 0.011575375 | 1.470220638 | 854.5692 | PC(22:6(4Z,7Z,10Z,13Z,16Z,19Z)/20:4(8Z,11Z,14Z,17Z)) | POS |
| M781 | 1.903233034 | 0.928452217 | 0.001648792 | 0.013763232 | 1.453941936 | 398.2124 | Methanone, [1-(cyclohexylmethyl)-1H-indol-3-yl](4-methoxy-1-naphthalenyl)- | POS |
| M376 | 1.977369594 | 0.983582553 | 0.002792947 | 0.019284434 | 1.39394111 | 284.2951 | Sph(d18:0) | POS |
| M39 | 1.514180473 | 0.598537168 | 0.005270207 | 0.029229684 | 1.353984553 | 152.0571 | Guanine | POS |
| M2454 | 1.415053626 | 0.500856728 | 0.011473675 | 0.049331159 | 1.289405314 | 738.5083 | PE(18:1(9Z)/18:4(6Z,9Z,12Z,15Z)) | POS |
| M41 | 1.422340869 | 0.508267253 | 0.017679936 | 0.065889344 | 1.245765601 | 253.0938 | Deoxyinosine | POS |
| M2118 | 1.667435197 | 0.737630693 | 0.028392663 | 0.089791482 | 1.278233468 | 752.5235 | PC(20:4(8Z,11Z,14Z,17Z)/14:1(9Z)) | POS |
| M2252 | 1.236314273 | 0.306045525 | 0.040649403 | 0.115779173 | 1.181078796 | 848.6168 | PE(22:4(7Z,10Z,13Z,16Z)/22:2(13Z,16Z)) | POS |
| M370 | 1.33451993 | 0.416320851 | 1.28E-06 | 0.000133365 | 1.460011513 | 140.0122 | O-Phosphoethanolamine | NEG |
| M110 | 1.81284621 | 0.858256542 | 4.20E-06 | 0.000250893 | 1.455728692 | 154.9754 | Phosphoglycolic acid | NEG |
| M259 | 1.441279684 | 0.527350322 | 9.93E-06 | 0.000422526 | 1.460279111 | 338.9902 | Fructose 1,6-bisphosphate | NEG |
| M54 | 2.468056073 | 1.303375172 | 3.59E-05 | 0.000891272 | 1.462793364 | 558.0655 | ADP-ribose | NEG |
| M62 | 1.304609094 | 0.38361759 | 0.000119672 | 0.001843134 | 1.419250313 | 407.2815 | Cholic acid | NEG |
| M158 | 1.121461396 | 0.165379959 | 0.000168537 | 0.002246358 | 1.413279294 | 487.1007 | Cytidine 5'-diphosphocholine (CDP-choline) | NEG |
| M245 | 1.534048352 | 0.617343956 | 0.000281265 | 0.00312405 | 1.403127365 | 115.004 | Fumaric acid | NEG |
| M223 | 1.274308578 | 0.349714673 | 0.000688819 | 0.005704181 | 1.37737276 | 193.0362 | Glucuronic acid | NEG |
| M83 | 1.20427297 | 0.268162441 | 0.000861462 | 0.006693219 | 1.386252608 | 383.115 | S-Adenosylhomocysteine | NEG |
| M249 | 1.538543664 | 0.621565388 | 0.001783367 | 0.010951578 | 1.344479709 | 266.0906 | Deoxyguanosine | NEG |
| M320 | 2.288179938 | 1.194200507 | 0.002775219 | 0.014638731 | 1.370213252 | 227.0676 | 2'-Deoxyuridine | NEG |
| M156 | 1.154196727 | 0.206889145 | 0.002840016 | 0.014865271 | 1.335756672 | 579.0293 | Uridine 5'-diphosphoglucuronic acid (UDP-glucuronate) | NEG |
| M107 | 1.219917296 | 0.286783344 | 0.003320573 | 0.016508497 | 1.373330806 | 259.0234 | Fructose 1-phosphate | NEG |
| M150 | 1.219917296 | 0.286783344 | 0.003320573 | 0.016508497 | 1.373330806 | 259.0234 | Fructose 6-phosphate | NEG |
| M151 | 1.219917296 | 0.286783344 | 0.003320573 | 0.016508497 | 1.373330806 | 259.0234 | Glucose 6-phosphate | NEG |
| M216 | 1.219917296 | 0.286783344 | 0.003320573 | 0.016508497 | 1.373330806 | 259.0234 | Mannose 1-phosphate | NEG |
| M78 | 1.219917296 | 0.286783344 | 0.003320573 | 0.016508497 | 1.373330806 | 259.0234 | Mannose 6-phosphate | NEG |
| M381 | 1.309832453 | 0.389382281 | 0.004252095 | 0.019663962 | 1.362634813 | 133.0148 | Malic acid | NEG |
| M362 | 1.108169263 | 0.148178257 | 0.007302703 | 0.029326061 | 1.303929476 | 96.9698 | Phosphate | NEG |
| M1450 | 1.168326278 | 0.224443231 | 0.007651741 | 0.030186362 | 1.24974936 | 445.0554 | CDP-Ethanolamine | NEG |
| M3145 | 1.25357022 | 0.326042813 | 0.010899876 | 0.038963924 | 1.220434645 | 239.0778 | Galactose | NEG |
| M488 | 1.105936375 | 0.14526839 | 0.011574246 | 0.040852857 | 1.218408484 | 117.0197 | Succinate | NEG |
| M92 | 1.401182073 | 0.486644435 | 0.020002666 | 0.060559722 | 1.294283749 | 243.0626 | Pseudouridine | NEG |
| M347 | 1.074287356 | 0.103379945 | 0.021966172 | 0.064795923 | 1.217348453 | 259.0233 | Galactose 1-phosphate | NEG |
| M2777 | 1.364550047 | 0.448425309 | 0.025388884 | 0.071852118 | 1.130758798 | 308.0821 | Indoxyl glucuronide | NEG |

**Continued Table S8:**

| **ID** | **foldChange** | **log2FoldChange** | **pvalue** | **FDR** | **vip** | **m/z** | **Name** | **type** |
| --- | --- | --- | --- | --- | --- | --- | --- | --- |
| M31 | 1.134815867 | 0.182458228 | 0.028542988 | 0.078543782 | 1.134280441 | 588.076 | GDP-fucose | NEG |
| M222 | 1.139438802 | 0.188323441 | 0.028932424 | 0.07937057 | 1.225647597 | 160.0623 | 2-Aminoadipic acid | NEG |
| M455 | 1.139438802 | 0.188323441 | 0.028932424 | 0.07937057 | 1.225647597 | 160.0623 | N-Methylglutamic acid | NEG |
| M936 | 1.096698392 | 0.133166818 | 0.029039686 | 0.07953237 | 1.126109559 | 73.0297 | Lactaldehyde | NEG |
| M328 | 1.298365582 | 0.376696662 | 0.031178841 | 0.083133675 | 1.135089001 | 686.1457 | Dephospho-CoA | NEG |
| M435 | 1.284218254 | 0.360890411 | 0.031526166 | 0.083718088 | 1.221745621 | 129.0198 | Mesaconic acid | NEG |
| M436 | 1.284218254 | 0.360890411 | 0.031526166 | 0.083718088 | 1.221745621 | 129.0198 | Itaconic acid | NEG |
| M442 | 1.378672971 | 0.463280282 | 0.031648442 | 0.08392609 | 1.108320279 | 165.0197 | Phthalic acid | NEG |
| M1207 | 1.240501014 | 0.310922915 | 0.040697399 | 0.100784801 | 1.074495312 | 161.0462 | Allose | NEG |

**Table S9 Significantly up‑regulated differential metabolites in positive and negative ion modes in BL group**

| **ID** | **foldChange** | **log2FoldChange** | **pvalue** | **FDR** | **vip** | **m/z** | **Name** | **type** |
| --- | --- | --- | --- | --- | --- | --- | --- | --- |
| M10 | 1.078407399 | 0.1089023 | 0.028628555 | 0.093304634 | 1.239397136 | 123.0554 | Nicotinamide | POS |
| M100 | 1.307134012 | 0.386407059 | 0.033865295 | 0.103975528 | 1.277199176 | 114.0663 | Creatinine | POS |
| M1560 | 1.554041356 | 0.636024898 | 0.045405193 | 0.126616555 | 1.123750988 | 816.592 | PC(22:6(4Z,7Z,10Z,13Z,16Z,19Z)/P-18:1(11Z)) | POS |
| M172 | 1.332738416 | 0.414393642 | 0.035693907 | 0.107376347 | 1.160572393 | 731.606 | SM(d18:1/18:0) | POS |
| M2118 | 1.817638106 | 0.862064986 | 0.016274155 | 0.064894534 | 1.311329288 | 752.5235 | PC(20:4(8Z,11Z,14Z,17Z)/14:1(9Z)) | POS |
| M2454 | 1.708266699 | 0.77253323 | 0.001833545 | 0.017023979 | 1.4596371 | 738.5083 | PE(18:1(9Z)/18:4(6Z,9Z,12Z,15Z)) | POS |
| M266 | 9.923110121 | 3.310792364 | 0.00560224 | 0.033579472 | 1.463676086 | 146.118 | Acetylcholine | POS |
| M2722 | 3.651728196 | 1.868579387 | 0.026854578 | 0.089549418 | 1.23263098 | 776.5112 | PC(18:3(9Z,12Z,15Z)/18:4(6Z,9Z,12Z,15Z)) | POS |
| M348 | 1.574292626 | 0.65470373 | 0.003811894 | 0.026393176 | 1.434117047 | 255.0986 | Nicotinamide riboside (NR) | POS |
| M471 | 1.488963908 | 0.574308784 | 0.012905081 | 0.056106605 | 1.353742348 | 444.0342 | Guanosine 5'-diphosphate (GDP) | POS |
| M51 | 2.482919251 | 1.312037343 | 8.81663607367946e-06 | 0.001736256 | 1.529728711 | 173.0215 | sn-Glycerol 1-phosphate | POS |
| M52 | 1.276501129 | 0.352194813 | 0.00433485 | 0.028580981 | 1.413469396 | 348.0709 | Adenosine monophosphate (AMP) | POS |
| M63 | 1.587294637 | 0.66656995 | 0.005715505 | 0.033971157 | 1.33850453 | 377.1466 | Riboflavin | POS |
| M76 | 6.283854007 | 2.651649663 | 0.048086925 | 0.13165987 | 1.240204955 | 399.1454 | S-Adenosylmethionine | POS |
| M81 | 1.208869823 | 0.273658896 | 0.027409714 | 0.090601093 | 1.303719615 | 241.0316 | Cystine | POS |
| M88 | 1.488144294 | 0.573514421 | 0.008174251 | 0.042252265 | 1.433732085 | 118.0865 | Betaine | POS |
| M107 | 1.151579694 | 0.203614255 | 0.009264393 | 0.035754618 | 1.289993072 | 259.0234 | Fructose 1-phosphate | NEG |
| M116 | 2.457970688 | 1.297467711 | 1.94E-09 | 5.42E-06 | 1.486694626 | 171.0068 | sn-Glycerol 3-phosphate | NEG |
| M124 | 1.257500968 | 0.330559511 | 0.004428596 | 0.021699151 | 1.31107944 | 191.0568 | Quinic acid | NEG |
| M127 | 3.130398005 | 1.646346096 | 0.000102229 | 0.001870195 | 1.473817834 | 283.0696 | Xanthosine | NEG |
| M1450 | 1.420121227 | 0.506014089 | 6.76E-05 | 0.001452128 | 1.447699718 | 445.0554 | CDP-Ethanolamine | NEG |
| M150 | 1.151579694 | 0.203614255 | 0.009264393 | 0.035754618 | 1.289993072 | 259.0234 | Fructose 6-phosphate | NEG |
| M151 | 1.151579694 | 0.203614255 | 0.009264393 | 0.035754618 | 1.289993072 | 259.0234 | Glucose 6-phosphate | NEG |
| M158 | 1.676685073 | 0.745611737 | 1.32E-07 | 3.50E-05 | 1.483401086 | 487.1007 | Cytidine 5'-diphosphocholine (CDP-choline) | NEG |
| M160 | 1.322478429 | 0.403244191 | 0.014562099 | 0.049226225 | 1.321261397 | 166.0152 | Quinolinic acid | NEG |
| M162 | 1.334968746 | 0.416805967 | 0.000682029 | 0.006472963 | 1.388425957 | 308.0999 | N-Acetylneuraminic acid | NEG |
| M190 | 1.43747892 | 0.523540799 | 0.000145723 | 0.002303557 | 1.460731289 | 426.0229 | 2'-Deoxyguanosine 5'-diphosphate (dGDP) | NEG |
| M20 | 1.494451178 | 0.579615767 | 0.000243686 | 0.003217346 | 1.416961059 | 203.0829 | Tryptophan | NEG |
| M216 | 1.151579694 | 0.203614255 | 0.009264393 | 0.035754618 | 1.289993072 | 259.0234 | Mannose 1-phosphate | NEG |
| M221 | 1.477981178 | 0.563627897 | 1.20E-07 | 3.38E-05 | 1.482102678 | 565.0512 | Uridine diphosphate glucose (UDP-glucose) | NEG |
| M222 | 1.740711734 | 0.799677309 | 3.19E-05 | 0.000930653 | 1.462658022 | 160.0623 | 2-Aminoadipic acid | NEG |
| M259 | 1.162078136 | 0.216707076 | 0.025465643 | 0.073901909 | 1.212870668 | 338.9902 | Fructose 1,6-bisphosphate | NEG |
| M261 | 1.417559024 | 0.503408807 | 5.40E-09 | 6.72E-06 | 1.486656474 | 346.0568 | 2'-Deoxyguanosine 5'-monophosphate (dGMP) | NEG |
| M275 | 1.477981178 | 0.563627897 | 1.20E-07 | 3.38E-05 | 1.482102678 | 565.0512 | UDP-galactose | NEG |

**Continued Table S9:**

| **ID** | **foldChange** | **log2FoldChange** | **pvalue** | **FDR** | **vip** | **m/z** | **Name** | **type** |
| --- | --- | --- | --- | --- | --- | --- | --- | --- |
| M2777 | 3.309720887 | 1.726709558 | 0.000184393 | 0.002677639 | 1.469001005 | 308.0821 | Indoxyl glucuronide | NEG |
| M278 | 2.973644969 | 1.572232411 | 6.54E-05 | 0.001427216 | 1.472315881 | 505.9902 | Adenosine 5'-triphosphate (ATP) | NEG |
| M279 | 2.973644969 | 1.572232411 | 6.54E-05 | 0.001427216 | 1.472315881 | 505.9902 | Deoxyguanosine triphosphate (dGTP) | NEG |
| M285 | 1.281804296 | 0.358176011 | 0.008432091 | 0.033596331 | 1.299734374 | 87.0089 | Pyruvate | NEG |
| M2942 | 1.534033755 | 0.617330228 | 0.030795786 | 0.084923553 | 1.187769259 | 267.0993 | L-Kynurenine | NEG |
| M2993 | 1.757258257 | 0.813326234 | 0.00021366 | 0.00295691 | 1.422969408 | 471.1052 | L-2-Aminoadipate adenylate | NEG |
| M2996 | 1.71479578 | 0.778036772 | 0.000505619 | 0.005270862 | 1.455640033 | 172.0621 | N-Acetyl-L-glutamate 5-semialdehyde | NEG |
| M31 | 1.709374025 | 0.773468105 | 6.02E-05 | 0.001361993 | 1.479366036 | 588.076 | GDP-fucose | NEG |
| M3145 | 1.217796581 | 0.284273167 | 0.031181617 | 0.085737284 | 1.1233329 | 239.0778 | Galactose | NEG |
| M3154 | 1.460058473 | 0.546026148 | 0.025320119 | 0.073613101 | 1.155618592 | 174.0777 | D-Proline | NEG |
| M337 | 1.562525454 | 0.643879692 | 0.003138269 | 0.017206692 | 1.419188589 | 604.074 | GDP-mannose | NEG |
| M342 | 2.893490702 | 1.532811008 | 0.003172736 | 0.017310895 | 1.381261614 | 147.0668 | Mevalonic acid | NEG |
| M36 | 1.152985307 | 0.205374129 | 0.011821474 | 0.042325944 | 1.253908357 | 180.0668 | Tyrosine | NEG |
| M370 | 1.298906984 | 0.377298122 | 1.22E-06 | 0.000133857 | 1.475460611 | 140.0122 | O-Phosphoethanolamine | NEG |
| M381 | 1.337065374 | 0.419070005 | 0.001879371 | 0.012466403 | 1.363606423 | 133.0148 | Malic acid | NEG |
| M4 | 1.093421549 | 0.128849714 | 0.014337421 | 0.04870199 | 1.27845124 | 124.0074 | Taurine | NEG |
| M413 | 2.13826372 | 1.096439797 | 0.028612008 | 0.080467231 | 1.222325505 | 165.056 | Desaminotyrosine | NEG |
| M455 | 1.740711734 | 0.799677309 | 3.19E-05 | 0.000930653 | 1.462658022 | 160.0623 | N-Methylglutamic acid | NEG |
| M483 | 1.406380403 | 0.491986873 | 0.000724019 | 0.006715922 | 1.441728086 | 141.0171 | 2-Oxoadipic acid | NEG |
| M488 | 1.202438432 | 0.265963027 | 0.00089309 | 0.007789883 | 1.38691505 | 117.0197 | Succinate | NEG |
| M54 | 5.292400829 | 2.403922331 | 1.21E-06 | 0.000133857 | 1.48513896 | 558.0655 | ADP-ribose | NEG |
| M62 | 3.13245822 | 1.647295267 | 2.34E-06 | 0.000194135 | 1.480816602 | 407.2815 | Cholic acid | NEG |
| M78 | 1.151579694 | 0.203614255 | 0.009264393 | 0.035754618 | 1.289993072 | 259.0234 | Mannose 6-phosphate | NEG |
| M79 | 1.429681295 | 0.515693577 | 2.23E-05 | 0.000753358 | 1.462889244 | 613.1411 | Cytidine monophosphate N-acetylneuraminic acid | NEG |
| M83 | 1.805626055 | 0.852499141 | 1.71E-06 | 0.000165183 | 1.47864589 | 383.115 | S-Adenosylhomocysteine | NEG |
| M936 | 1.113004784 | 0.154459794 | 0.016767062 | 0.054698144 | 1.200129297 | 73.0297 | Lactaldehyde | NEG |
